# Open problems in human trait genetics

**DOI:** 10.1186/s13059-022-02697-9

**Published:** 2022-06-20

**Authors:** Nadav Brandes, Omer Weissbrod, Michal Linial

**Affiliations:** 1 School of Computer Science and Engineering, The Hebrew University of Jerusalem, Jerusalem, Israel; 2 Department of Epidemiology, Harvard T.H. Chan School of Public Health, Boston, MA USA; 3 Department of Biological Chemistry, The Alexander Silberman Institute of Life Sciences, The Hebrew University of Jerusalem, Jerusalem, Israel

**Keywords:** Statistical genetics, Human phenotypes, Complex human traits, GWAS, Genome-wide association studies, PRS, Polygenic risk scores, Heritability, Missing heritability, Population structure, Diversity, Rare variants, GxG, Epistatis, Non-additive genetic effects, GxE, Gene-environment interactions, Linkage disequilibrium, Causal variants, Recessive effects

## Abstract

Genetic studies of human traits have revolutionized our understanding of the variation between individuals, and yet, the genetics of most traits is still poorly understood. In this review, we highlight the major open problems that need to be solved, and by discussing these challenges provide a primer to the field. We cover general issues such as population structure, epistasis and gene-environment interactions, data-related issues such as ancestry diversity and rare genetic variants, and specific challenges related to heritability estimates, genetic association studies, and polygenic risk scores. We emphasize the interconnectedness of these problems and suggest promising avenues to address them.

**Supplementary Information:**

The online version contains supplementary material available at 10.1186/s13059-022-02697-9.

## Preface

Since the days of Gregor Mendel and his series of pea plant experiments in the middle of the 19th century, a key motivation of genetic research, perhaps the leading motivation, has been to understand the link between genotype and phenotype. When the human genome project was declared complete in 2003, hopes were high for a full understanding of human genetics and its effects on human traits. The idea to genotype large cohorts of individuals and, through simple statistical tests, compiling an atlas mapping between genes and human traits—be it diabetes, schizophrenia, or height—had been around for quite some time [1]. But excitement rapidly shifted to disappointment, as efforts to find the genetic variation underlying complex human diseases ended up explaining only a small fraction of the phenotypic variance [2].

Almost two decades later, with millions of individuals genotyped across thousands of genome-wide association studies, it is now well acknowledged that things are not that simple. But it is worth asking, why are they not, actually? Why have not we mapped most of the genetic variation underlying human traits, and why are we still unable to make accurate individual phenotypic predictions from genetic data? What are the concrete problems we are now facing, and what bottlenecks are slowing us down and preventing genetic research from unlocking its full potential? Asking these questions and attempting to answer them will allow us to make more effective progress and eventually achieve the field’s long-term potential.

## Scope

The domain of knowledge we are dealing with, mapping and understanding the genetic variation underlying human phenotypic variation, is a huge area of scientific inquiry. Among its main subdomains are as follows: (i) genetic association studies, which seek causal links between genetic elements and human traits [2–4], (ii) polygenic risk scores, which aim to predict traits from genetics [5], and (iii) heritability estimates, which estimate the fraction of a trait’s variance that is due to genetic variation [6].

The primary applications of these research activities are twofold: (i) obtaining insight into the biological mechanism at the molecular and cellular level underlying the disease or trait under study, and (ii) making informed predictions that can be clinically useful, even in the absence of knowledge about mechanism or causality. Even if we do not understand why an individual is at high risk for heart disease, it is still useful to know that they are at such risk, especially if we can reliably quantify it [7].

This review only deals with open problems directly related to the interface between genetics and human traits. Topics related to genetics but not to phenotypic variation in present-day humans (such as functional elements in the human genome, or questions of evolution, conservation, and fitness) are out of our main focus. We also do not discuss methods that use genetics only as an instrument to study relationships between traits, such as Mendelian Randomization (which attempts to find out through genetic evidence whether one trait affects another, for example whether high cholesterol levels increase the risk for heart disease) [8]. Likewise, we do not delve into technical aspects of genotyping and sequencing (including variant calling and quality control) and cancer genomics (i.e., the study of somatic mutations in tumors). Also outside of our main focus are gene expression, epigenetics, and other omics.

It is common to distinguish between Mendelian and complex traits. Mendelian traits are traits that follow Mendel’s laws of inheritance, in particular the law dictating pure dominant or recessive inheritance with high penetrance. A Mendelian trait is typically linked to a single gene (sometimes a handful of genes), and it tends to be deterministically determined by genetics alone. Non-Mendelian traits, known as complex traits, show the opposite characteristics: they are typically influenced by variants across numerous (sometimes thousands) genomic loci, each with limited effect, and they typically exhibit substantial environmental effects and tend to be nondeterministic. While we generally understand the genetics of Mendelian traits [9], the study of complex traits still poses many challenges.

This review primarily focuses on analytical challenges in studying the genetics of complex human traits. Because we are interested in methodology, we mention specific traits only as examples and illustrations of general trends, and while we focus on humans, it is useful at times to gain insight from the study of other organisms.

## A list of open problems

The tradition of publishing “open problems” is borrowed from the mathematical disciplines, where the explicit discussion of major open challenges has a great role in prioritizing work, sub-branching, and otherwise advancing these fields. This review is an attempt to map concrete and solvable open problems related to the genetics of human traits.

Altogether, we have identified 16 major open problems which we consider important to the field (Table 1). Since these open problems are highly interconnected, we go through them in the remainder of this review by thematic topics (rather than by the categorical ordering in Table 1).

**Table 1:** Open problems

Category	#	Open problem	Brief explanation	Why it is important	Related open problems	Selected references
**General**	**1**	**Population structure**	Genetic studies are confounded by the ancestries of participants. Mounting evidence points towards residual population structure not accounted for, while overcorrection can obscure genuine genetic signal.	Without resolving this, it will be difficult to trust the results of genetic studies.	4, 6, 7, 12, 16	[10, 11]
	**2**	**Non-additive and epistatic genetic effects (GxG)**	The assumption that phenotypes can be approximated by summing separate genetic effects is ubiquitous in genetic studies. If incorrect, this could undermine many results. Also, how do we identify and quantify epistatic effects?	Our ultimate goal is an accurate genetic model of human traits, linear or not.	11, 14	
	**3**	**Gene-environment interactions (GxE)**	Genetic effects may be contingent on environmental conditions. Such interactions are difficult to discover, and their overall contribution to phenotypic variance is not clear. Substantial GxE interactions would also undermine many methods.	GxE interactions are potentially an important piece in the genetic puzzle, which can highlight the mechanism of genetic associations and inform interventions.	11, 13	[12]
**Data**	**4**	**Rare variants**	Most genetic studies of complex traits deal only with common variants, even though the strongest effects are expected in rare variants. In aggregate, they may contribute substantially to heritability. Key challenges are lack of statistical power and genotyping.	Rare variants may be important to many complex traits. Neglecting them would leave us with an incomplete understanding of the genetic variation underlying these traits.	1, 5, 11	
	**5**	**Non-standard genetic variation**	Routine pipelines are optimized for simple variants (i.e., single-nucleotide variants and small indels), while commonly overlooking more complex genetic variation, including structural variants, copy number variation, repetitive regions and variants on the X, Y or MT chromosomes.	These types of variants contribute substantially to many traits.	4, 11	
	**6**	**Family-based vs. population-based cohorts**	Family-based study designs naturally overcome many challenges of cohort studies, specifically with respect to population structure, environmental biases, and direct vs. indirect genetic effects. However, family-based genetic resources are scarce, and there are not enough methods to analyze them.	Family-based genetic data could play an important role in studying genetic effects, especially when causality is sought.	1, 12	[13]
	**7**	**Ancestry diversity**	Individuals of non-European ancestry are heavily underrepresented in genetic datasets, leading to inequality in access to medical knowledge. More diversity would also help deal with population structure and establish the causality of genetic associations.	We are interested in understanding the genetics of all of humanity, and we cannot afford to discard such a powerful tool.	1, 12, 16	[14]
	**8**	**Phenotype definition**	Studied traits are often not entirely well defined, and there is often a lot of noise in the phenotyping process (mostly with respect to binary phenotypes).	Noisy and biased data hinders our progress.		[15]
	**9**	**Selection bias**	Genetic associations may reflect people’s decision to participate rather than the studied phenotype.	This is potentially a major source of bias.		
**Heritability**	**10**	**Heritability estimate interpretation**	It is not entirely clear what the “correct” way to define and measure heritability is and how heritability estimates should be interpreted. For example, do they provide an upper bound on the predictive power of polygenic risk scores?	Heritability estimates provide a lot of insight and guide our progress, and they could be even more useful if we reached a consensus on what they mean exactly.	11, 14	
	**11**	**Missing heritability**	This is a classic problem, asking why detected associations explain only a small part of the heritability in most complex traits, and why there is a large gap between heritability estimates obtained from SNP-based and twin-based methods. Despite a lot of progress in suggesting solutions and collecting evidence, the problem is still not fully resolved.	As long as this is not fully resolved, there are lingering doubts that our understanding of genetic effects is flawed in some fundamental way.	2, 3, 4, 5, 10, 14	[16, 17]
**Association studies**	**12**	**From association to causality**	Most genetic associations implicate entire genomic regions, and it is considered a hard problem to pinpoint the exact causal variants. It is also important to rule out confounding and other statistical biases.	If we want to learn from genetic associations, we need to be able to detect causal variants and genes.	1, 6, 7	[18]
	**13**	**From causality to mechanism**	Even after the causality of genetic elements is established, understanding the molecular mechanisms behind them is a grand challenge. To date, only a very small fraction of genetic discoveries are understood at that level.	Without understanding the mechanism of genetic associations, they provide only limited biological and medical insight.	3	
**Polygenic risk scores**	**14**	**Genotype-to-phenotype prediction performance**	Our ability to make accurate phenotypic predictions from genetic data is still very limited, even in highly heritable traits. Other than increasing sample sizes, we do not have very effective strategies to improve predictions.	Accurate genotype-to-phenotype predictions have an enormous clinical potential.	2, 10, 11, 15	[5]
	**15**	**The clinical utility of polygenic risk scores**	The use of polygenic risk scores in the clinics remains quite limited. To be clinically useful, predictive models need to be proven robust and reliable.	If successfully implemented in the clinics, these models have the potential to revolutionize healthcare and usher in the era of personalized medicine.	14, 16	[7, 19]
	**16**	**Model transferability**	Polygenic risk scores trained in one setting generally do not generalize well to other settings, including different ancestries or genotyping technologies.	This is critical for ensuring the robustness of these models and allow them to be used in the clinics, and for their fruits to benefit all groups.	1, 7, 15	

## Genetic association studies and their limitations

Genome-wide association study (GWAS) [2–4] is the most common type of genetic study. The idea behind GWAS is straightforward: independently examine each genotyped variant in the genome, and test it for statistical association with the studied phenotype. To minimize confounding by non-genetic factors (such as age), statistical testing is typically done with variations of linear or logistic regression (depending on whether the studied trait is continuous or binary, respectively). The main strengths of GWAS are its simplicity and generality. Since the method makes almost no assumptions about the nature of associations and their biological basis, GWAS can in principle identify any genetic association, provided a sufficiently large cohort. There are, however, certain obstacles limiting one from inferring a causal link in the face of a significant GWAS association. The main issues specific to genetic studies are population structure and linkage disequilibrium

### Population structure

**Fig. 1:**
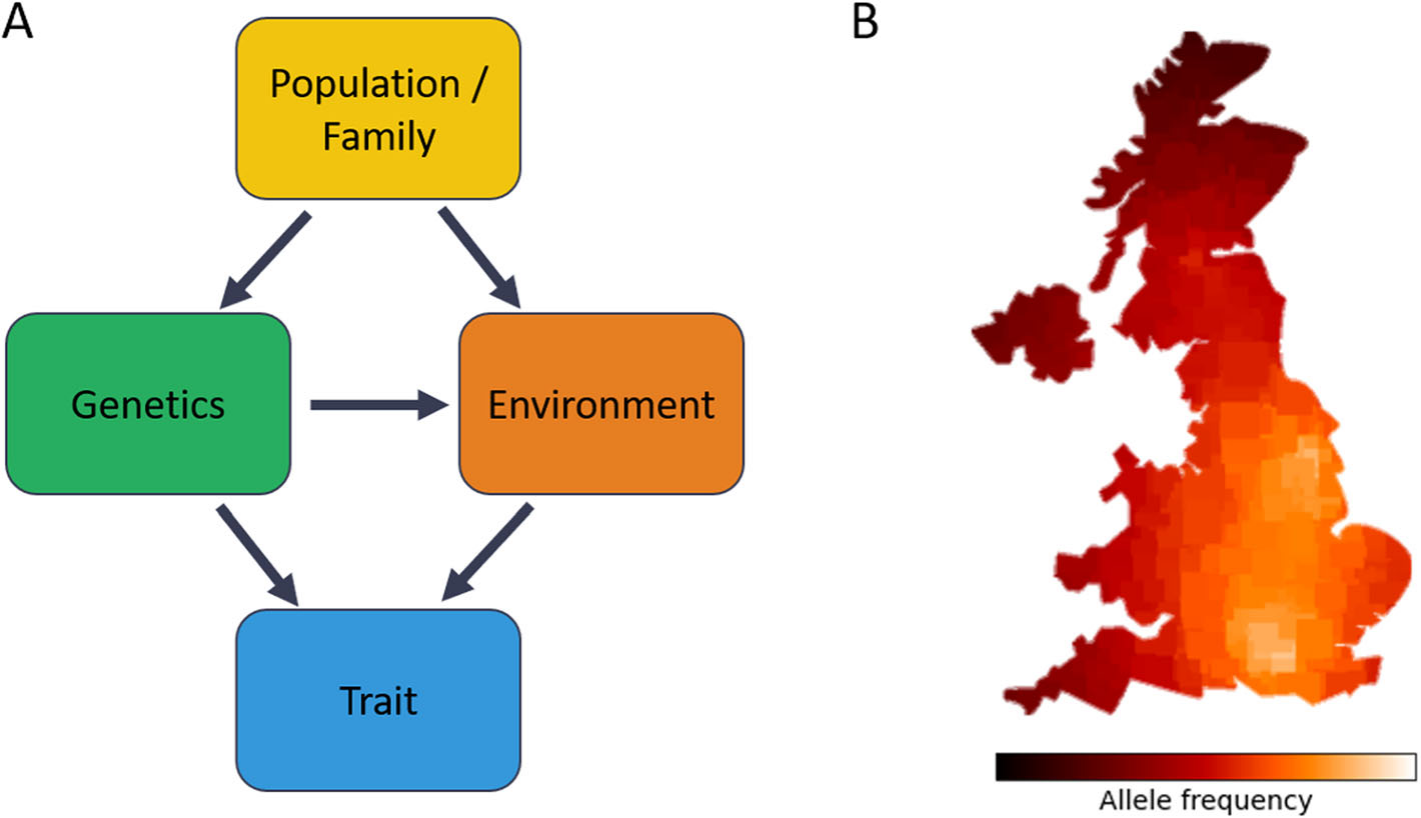
Population structure confounds human genetic studies. **A** The population that an individual is born into influences their genetics and their environment, which are the two components affecting traits. As a result, genetic associations with human traits are confounded by population structure. **B** Even when considering a specific human group and controlling for the major axes of genetic variation in a cohort, the allele frequency of some variants can still vary across populations and exhibit clear geographic patterns, a problem known as “residual population structure”

The phrase “correlation does not imply causation” needs no rehearsing. In the case of genetics, traits only rarely affect genetics (e.g., through assortative mating [20]). This means that if we seek to draw a causal genotype-to-phenotype link based on a statistical association, our primary concern is confounding, namely the existence of other variables affecting both an individual’s genetics and the trait (there is also the less trivial concern of collider bias, which we address later). Specifically, we know that an individual’s genetics is determined by the genetics of their parents. It follows that the precise family and ancestry of an individual (generally simply referred to as their “population” or “ancestry”) is a suspect confounder for any genetic association, as it affects the environment which an individual is born into (e.g., weather, nutrition) and, as a result, can affect the studied trait (Fig. 1A) [21].

To avoid spurious associations, genetic studies must account for population structure. The most common method for that is principal component analysis (PCA) [11]. It was shown that accounting for the 5–40 top principal components of genetic variation in a cohort (by including them as additional covariates in the regression analysis) can eliminate spurious results due to population structure. More principled guidelines for which principal components should be included have also been suggested [22]. Additionally, it is a common practice to split cohorts by self-reported ethnic identities [14]. Another class of commonly used methods are linear mixed models, which can control for even more nuanced population structure [23, 24].

While it is crucial to account for population structure, overcorrection could lead to reduction of statistical and predictive power, and to underestimation of the role of genetics in traits. Focusing on homogenous ancestry groups also underplays the potential genetic basis of phenotypic differences between groups and excludes a large proportion of humans with more complex ancestries.

Despite the capacity of existing methods to account for most of the phenotypic variance that is due to population structure, there are increasing concerns that residual population structure can still lead to spurious associations, especially with very large cohorts that are now emerging (open problem #1: population structure; Fig. 1B) [10]. For example, unaccounted population structure led researchers to mistakenly conclude that height-associated genetic variants were under selection in the European population [25]. It is still not fully understood how residual population structure affects the results of GWAS and polygenic risk scores. More research is also needed for establishing new methods and practices to address the problem. Two strategies seem particularly promising: (i) studying more diverse populations (especially non-Europeans) and (ii) using family-based designs for genetic studies.

The absence of diversity in genetic studies is hard to overemphasize (open problem #7: ancestry diversity). Individuals of European ancestry are overwhelmingly overrepresented in contemporary cohorts and, as a result, most published results do not directly transfer to other ancestry groups [26]. The immediate implication is that many of the benefits of genetic studies are of little benefit to most of the world’s population. Moreover, studies that are constrained to only one ancestry group may suffer from residual population structure driven by subpopulations of that ancestry group (e.g., across different geographic regions in Europe) [10]. Hence, ancestry diversity is also an incredibly powerful strategy for avoiding spurious associations driven by population structure [14]. The reason is that, while it is entirely possible for a variant to be spuriously correlated with a trait within one ancestry group due to specific subpopulations, it is unlikely to observe the same trend in an entirely different ancestry group. Thus, the replication of a genetic association across multiple ancestries is strong evidence for causality. In addition to more diverse datasets and increased awareness by researchers, there is also need for better analytical methods for robust analysis of cross-ancestry cohorts [14].

Another promising strategy to account for population structure is family-based studies [13]. As an illustration, consider the case of trios, where the two parents of each studied individual are also genotyped. When testing an autosomal variant, one can focus on trios where at least one of the parents is heterozygous for the tested variant. According to Mendel’s laws of heredity, the child is then expected to have exactly 50% chance of inheriting the variant from that parent and, crucially, this happens completely at random. By testing the fraction of transmitted variants among cases and controls (or, in the case of a continuous phenotype, the correlation between the phenotype and the frequency of transmitted variants), one can establish a robust association. Since Mendelian transmission is random, once the parents’ genetics is accounted for, this study design avoids the problem of population structure altogether. Like trios, more complex family structures can also be utilized in similar ways [13]. On the downside, families are harder to recruit than unrelated individuals. In addition, each trio comprises only a single datapoint, meaning that the effective sample size is only a third of the number of genotyped individuals.

While these days family-based studies are mostly used in the context of Mendelian traits, we argue that they should play a greater role in the study of complex traits [17, 27]. It is becoming increasingly evident that certain questions in human genetics can be decisively answered only through this study design (Table 1). Accessible resources with an abundance of related individuals, on the scale of contemporary large-scale biobanks [28, 29], with rich genotypic and phenotypic data, could be a major force advancing the field. Unfortunately, such resources are still scarce (open problem #6: family-based vs. population-based cohorts). Contemporary family cohorts are not easily accessible and are typically restricted to specific phenotypes (e.g., [30]). Also, methods combining and extracting signal from both family- and population-based data could be highly beneficial to the field. For example, an analysis of individuals from the UK Biobank, some of which are family relatives, showed an increase in statistical power compared to a filtered dataset of unrelated individuals (beyond the increase attributed to the larger sample size) [31].

### Linkage disequilibrium and the pursuit of causal variants

Linkage disequilibrium (LD) is the phenomenon that, due to inheritance through homologous recombination, individuals with a specific allele of one variant are likely to have a specific allele of a neighboring variant. A combination of multiple alleles that appear together in an individual across neighboring variants in a genomic region is often referred to as a haplotype. The resulting LD structure of correlated variants and haplotypes has profound implications for genetic studies [3].

In some ways, the phenomenon of LD is quite convenient. LD allows genotype imputation, namely the deduction of an individual’s genotype across a large set of genetic variants from a much smaller set of variants. Genotype imputation relies on haplotype reference panels which provide the full haplotypes of a large cohort of individuals (e.g., 32,488 individuals in the Haplotype Reference Consortium [32] or 97,256 in TOPMed [33]). Like most genetic resources, haplotype reference panels and DNA microarrays are still somewhat biased towards individuals of European ancestry (although improvements have been made [33]). As a result, the genetic coverage and quality of genotype imputation are typically lower for non-Europeans (open problem #7: ancestry diversity) [14]. Additionally, the common use of external haplotype reference panels to account for LD in published GWAS results (e.g., to conduct a meta-analysis) yields biased estimates of the actual LD patterns in the original dataset and negatively affects downstream analysis [34]. Publishing the full pairwise LD matrices together with the rest of the GWAS summary statistics could be a beneficial norm [35].

Due to LD, genetic variation has far fewer degrees of freedom than would naively be assumed by the total number of variants in the human genome. Because of that, adjusting the *p*-values that are independently derived for each tested variant in GWAS using Bonferroni correction would be too conservative. Instead, it is assumed that the LD structure in human is such that an individual’s genotype has roughly 1M degrees of freedom, meaning a genome-wide significance threshold of 5E−08 (or an exome-wide significance threshold of 5E−07, derived from roughly 100K degrees of freedom). While these thresholds are the norm in genetic studies, more stringent cutoffs have been recommended under certain conditions [36].

**Fig. 2:**
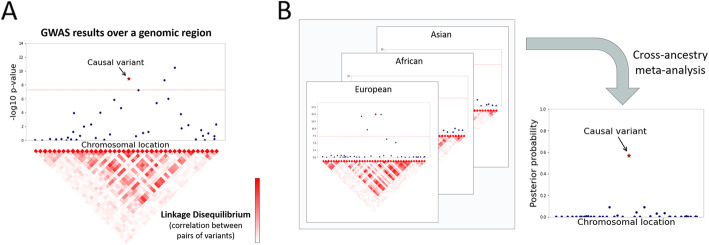
Identifying causal variants in the presence of linkage disequilibrium. **A** A single causal variant is in linkage disequilibrium with other nearby variants. As a result, variants that are correlated with the causal variant also obtain significant *p*-values even though they are not causal. **B** Combining GWAS summary statistics from three different ancestry groups, each exhibiting a different linkage disequilibrium pattern, to fine-map the results. By assuming that only one of the variants is causal, it can be recovered with high confidence

A negative consequence of LD is its obscuring of causal variants. Even when a genomic locus is robustly established as causally linked to a phenotype, it is very difficult to tell with certainty which of the variants in that locus are behind that causal link, a task known as fine-mapping (Fig. 2A). Numerous fine-mapping methods have been developed [34, 37–39]. These methods often appeal to Bayesian reasoning and rely on functional genomic annotations, under the assumption that causal variants are more likely to be located in functional sites of the genome. Despite these efforts and the great progress made, it is still an open problem how to establish the causality of a variant or a gene with certainty (open problem #12: from association to causality). In addition to method development, there is much room for the formalization of standards for establishing causality for research or clinical purposes [40].

Cross-ancestry genetic studies can be very useful for fine-mapping, in a similar way to their utility in dealing with population structure (open problem #7: ancestry diversity). Since different populations show different LD structures, a cross-ancestry meta-analysis will generally point at a much smaller set of candidate causal variants within a genomic region (Fig. 2B) [4, 14, 37]. However, if a genetic effect is unique to a specific population (e.g., due to interactions with other genetic or environmental factors), cross-ancestry analysis will not fine-map it effectively.

Another useful approach to pinning down the causal elements underlying genetic associations is to abandon the pursuit of specific variants and shift our attention to larger elements of the genome such as genes and pathways. Gene-based methods have become increasingly popular in recent years [41–45]. For example, there are methods detecting genes that are affected by variants influencing their expression to different levels in diseased cases compared to healthy controls [43, 44]. Among the merits of gene-based methods are reduced burden of multiple testing, easier interpretation of associations, and the ability to aggregate signal spread across many variants. If distinct variants that are not in LD perturb the same gene (e.g., change its coding sequence or expression level) and are associated with the same trait, then this could comprise an overall stronger evidence for the causality of that gene. However, gene-based approaches are still susceptible to LD; it is possible that signal from nearby variants would leak into variants affecting the gene. Moreover, even in the absence of LD, the expression of genes is often correlated, meaning that the association between the phenotype and the expression of a causal gene could leak into other genes. Gene-based methods are also sensitive to modeling assumptions and the specific details of how they aggregate the signal spread across variants.

An important factor limiting our progress towards established standards for causality is the shortage of confirmed, experimentally validated causal variants that can be used as a gold standard for validation and evaluation of methods. As a result, evaluation of methods in the field is typically based on computational simulations (which are sensitive to modeling assumptions). In addition to empirical benchmarks, the research of causal variants could benefit from well-designed competitions (similar to the competitions held by the protein research [46, 47] and genome interpretation [48] communities).

### Direct vs. indirect effects

Even when a variant or a gene is proven causal, its phenotypic effect might be indirect. Direct genetic effects influence traits within the individuals who carry the causal alleles, while indirect genetic effects influence their relatives. Important types of indirect genetic effects include (i) parental effects (that influence traits in children through their parents), (ii) assortative mating (i.e., influences on mate choices, which in turn can lead to further parental effects), and (iii) sibling effects (which may also influence traits through the environment). While both direct and indirect genetic effects are causal, the distinction between them is often crucial. For example, when seeking drug targets to treat afflicted individuals, only direct genetic effects are expected to be relevant. Common analytical methods such as GWAS do not distinguish between direct and indirect genetic effects, and it is still very much an open challenge to effectively dissect the two. Family-based studies that control for parental genotypes, on the other hand, in addition to being useful for separating causal from confounding effects, can be used to further separate causal effects into direct and indirect effects (open problem #6: family-based vs. population-based cohorts) [18].

### From causality to mechanism

Even among genetic effects for which causality is established, only a small fraction have a known, well-established biological mechanism (open problem #13: from causality to mechanism) [49, 50]. As most causal variants are in non-coding regions and exert weak effects, it is challenging to understand how they affect traits [4]. Without understanding how a variant affects a disease, it is of little use in providing biological insight on its etiology and progression.

At the beginning of the GWAS era, researchers were struggling to identify robust associations. It appears that nowadays we have the opposite problem: we are flooded with hundreds of thousands of genetic associations that we do not really know what to make of [4]. It was actually suggested that many complex traits are not only polygenic (i.e., genetically driven by many genes), but could in fact be considered omnigenic, namely that they are affected by most of the genes in the genome [51]. While the term has also been used with reference to a specific mechanistic model suggested for such extreme polygenicity, here we use “omnigenic” simply to refer to extremely polygenic traits, irrespective of the mechanism. For example, it was estimated that more than 71% of the 1 Mbp regions in the human genome contain at least one schizophrenia risk variant [52]. In such cases, additional mapping of genomic loci associated with omnigenic traits, without considering other factors (such as effect sizes), is not expected to substantially contribute to our understanding of mechanism.

One limitation in establishing causality and discerning mechanism that is specific to human genetics is our inability to run controlled experiments, which are an indispensable tool in plants and animals. There are however several strategies to utilize experimental methods in human genetic studies. One approach is to validate human genetic associations in other model organisms (e.g., by knocking out a homologous gene and observing the phenotypic effects on the animal). Another strategy is to look at cellular and tissue phenotypes for which experiments can be conducted as a proxy for the studied phenotype (e.g., using primary culture, human cell lines, or induced stem cells). We now possess high-throughput experimental methods that can not only functionally annotate fixed features of the genome (such as introns and promoters), but also detect the variable effects of genetic variation. For example, Perturb-seq, a recently developed method combining genetic perturbations with single-cell RNA sequencing, can detect variants that causally affect gene expression [53, 54].

Using such experimental methods, we can observe the effects of genetic variants on cells and postulate on the mechanism of their effects on human traits. Functional genomic annotations derived from experiments (and curated in resources such as ENCODE [55], GTEx [56], or HCA [57]) are also useful for suggesting mechanistic interpretations. Unfortunately, experimental methods are still dramatically more expensive (in time, labor and money) than analytical methods. They also force additional decisions such as which cell types to study. Moreover, as many complex diseases and conditions are manifested at the organism level, without known cellular-level indicators, cell-based methods could be inadequate. In light of these challenges, devising experimental and analytical strategies for finding or guiding the search for biological mechanism would be of tremendous benefit.

The phenomenon of pleiotropy, where the same genetic factor affects multiple traits, can also complicate the study and discussion around the mechanism of genetic effects. For example, a variant may indirectly affect a trait through its influence on another trait (not to be confused with indirect genetic effects on other individuals, as discussed in the previous chapter) [58].

## Polygenic risk scores: unfulfilled potential

Considering the limitations of GWAS and the fact that even variants that are proven causal tend to exert only weak effects, raw GWAS results provide limited utility for predicting complex traits (e.g., to detect individuals who are at high risk of developing type 2 diabetes). To make a meaningful prediction about a complex trait, one has to aggregate the weak signals spread across many genomic regions. An analytical model aggregating an individual’s genotype into an overall phenotypic prediction is called a polygenic risk score (PRS) [5]. In essence, this can be seen as a machine-learning task: training a model that takes an input *x* (one’s genotype, and optionally other variables) and outputs a prediction *y* (the individual’s predicted trait value). It should be noted that some define PRS as strictly linear models (which aggregate variants by multiplying them with learned weights and summing the multiplication terms), but we prefer to define PRS more generally as any genotype-to-phenotype predictive model.

PRS can be trained in various ways. Given a sufficiently large cohort of genotyped and phenotyped individuals, one can train a PRS from scratch using standard machine-learning algorithms on individual-level data. More commonly, summary statistics of published GWAS results are meta-analyzed into a linear model [5].

Accurate and reliable PRS have the potential to transform healthcare. Many common diseases (including metabolic, psychiatric, autoimmune, and cardiovascular diseases) have a very substantial genetic component [59]. Knowing that individuals are at elevated or reduced risk for a specific disease could prioritize screening and follow-ups, guide diagnosis, and inform medical interventions [7]. Unlike most clinical factors, genetic factors do not change throughout one’s lifespan (although their interactions with environmental factors may). It follows that a person’s genetics needs to be measured only once, and potentially provide many different PRS (for multiple traits) simultaneously. PRS could even update automatically as models improve.

However, despite over a decade of refining models with exponentially increasing cohort sizes, the predictive power of most PRS is still quite poor (open problem #14: genotype-to-phenotype prediction performance). While there has been some success in genetic prediction of specific phenotypes (such as height [60]), most diseases and clinically relevant traits are still far from the full potential of genetics-based risk assessment; the phenotypic variance explained by existing models is only a small fraction of the trait heritability, and most models do not reach clinical relevance [7]. Many of the potential reasons for the unsatisfying performance of PRS are linked to the question of missing heritability and to questions surrounding nonlinear genetic effects, which are addressed later. It remains an open question whether better methodology would allow us to substantially improve PRS performance without larger cohorts. A useful practical approach to improving PRS performance is to incorporate clinical factors into the predictive models on top of genetic markers (e.g., using body mass index and birth weight to improve type-2-diabetes PRS [61]). However, as of today, PRS-based risk assessment generally provides only marginal benefit on top of clinical predictors already used.

Another problem with PRS is in their capacity to generalize from the cohort they have been trained on to other settings, including different populations and genotyping technologies (open problem #16: model transferability). This is one of the main barriers for deploying these models in real-world clinical settings. In PRS, it is usually not necessary to pin down the causal variants (as non-causal variants that are only in LD with the causal variants can provide the same predictive power), but this may negatively affect model transferability due to population-specific LD patterns [19, 26]. Other dissimilarities between populations that can limit model generalization include differences in allele frequencies or effect sizes, or different interactions of the genetic effects with environment or other genetic factors. Like with many other problems in genetic research, here too it is extraordinarily helpful to use cross-ancestry data when training or fine-tuning PRS (open problem #7: ancestry diversity) [14]. When a PRS is transferred from one dataset to another, even when both are in principle of the same population, substantial declines in performance are still common [62]. The instability of PRS is often attributed to batch effects and to differences in genotyping technologies across datasets, but we still lack a good understanding of the reasons for these sensitivities. Even within the same dataset and ancestry group, prediction accuracy can vary based on characteristics such as sex, age, or socioeconomic status [63]. At present, we do not have a good theoretical framework estimating how much accuracy loss we should expect when transferring PRS between settings. Even more importantly, there is a great need of methods and strategies that would make PRS more robust and reliable. Such strategies could include adjustment and calibration of models to new settings, or training them to be more robust in the first place. Perhaps the ongoing revolution of causal inference could play a role in training PRS that capture causal signals instead of merely statistical associations which, in addition to many other benefits, would be more robust [64].

## Genetics in the clinics: are we there yet?

Genetic tests are routinely used in many clinical settings, including parental screening, diagnosis of children with developmental disorders, and drug prescription [40, 65]. To diagnose Mendelian traits, genetic counselors look for pathogenic variants that explain the observed phenotype and clinical history of the patient. However, a known variant with affirmed pathogenicity is not always found. For example, the Mendelian disease could be the result of a rare or de novo variant not previously reported. In the absence of conclusive pathogenic variants, genetic counselors may resort to more circumstantial evidence, such as the predicted functional effects of variants based on computational algorithms. The American College of Medical Genetics and Genomics recommends a five-tier system of classification for variants relevant to Mendelian disease, based on the strength of evidence supporting their role in the disease: (1) pathogenic, (2) likely pathogenic, (3) uncertain significance, (4) likely benign, or (5) benign [40].

Another potential use of genetics is in drug prescription. Pharmacogenetics (sometimes referred to as pharmacogenomics) studies genetic variation underlying individual response to medication, which could be used to predict (i) individual drug dose, (ii) absence of response to a drug, or (iii) individuals at serious risk of toxicity if a drug is prescribed. For example, it is now a routine clinical protocol to test for the HLA-B*57:01 allele before prescribing the anti-HIV drug abacavir, which could lead to hypersensitivity reactions in carriers of this allele. However, current uses of pharmacogenetics as part of standard medical care are still limited to a small number of well-established associations [65].

In contrast to its immense utility for Mendelian traits, genetics is rarely used as part of routine clinical practice when dealing with complex traits. This is an enormous unfulfilled opportunity, since complex diseases comprise most of the burden of diseases in developed countries, and there is a lot to gain from early intervention. The GWAS literature reporting on over 200,000 genotype-phenotype associations is almost never directly used by clinicians. Clinical practice calls for a much stronger burden of proof, and the weak effect sizes associated with most GWAS results do not generally justify substantial deviations from routine medical care. Some exceptions exist, mostly related to cancer predisposition (e.g., testing variants in BRCA1 and BRCA2 to screen for breast and ovarian cancer [66]).

Unlike raw GWAS results, PRS have the potential to revolutionize healthcare. But, as of today, PRS are not yet adopted in clinical settings in a meaningful scale [7, 19]. To be accepted by the medical community, the clinical utility of PRS should first be established (open problem #15: the clinical utility of polygenic risk scores). Part of the problem is the aforementioned issues of PRS having, for the most part, limited predictive power (open problem #14: genotype-to-phenotype prediction performance) and generalizability (open problem #16: model transferability). On top of that, proving the reliability and robustness of PRS-guided protocols over classic medical protocols is a complex process which could require randomized-controlled trials. Expensive clinical trials are mostly required in cases where risky policy changes are attempted (for example, delaying mammographic screenings beyond the recommended age for low-risk women). When genetics is only used to take extra precautions (for example, undergoing colonoscopy screening earlier in life than would otherwise be recommended), there is usually no need for full clinical trials, but health care providers would still want to see good evidence that the decision is sensible and cost-effective. When considering the clinical utility of PRS, it should be kept in mind that they need not provide clear predictions for all or even most individuals. Identifying a subset of individuals who are at elevated or reduced risk for disease is sufficient in many settings [7]. Above all, PRS should be seen as a supplement, not replacement, for traditional risk prediction models [19].

In the long run, genetic studies (and PRS in particular) could have additional clinical applications, for example in screening human embryos for complex diseases or other polygenic traits (which raises both ethical and practical concerns) [67, 68].

## Heritability estimates and controversies

### What is heritability and why is it important?

Human genetics literature is filled with estimates of heritability for various human traits based on a variety of methods. For example, a recent study on the heritability of 551 complex traits (based on the UK Biobank) estimated height to be around 70% heritable and fluid intelligence score around 25% in the white British population [59]. Another study estimated the heritability of schizophrenia at 80% (based on the Nationwide Danish Twin Register) [69]. But what do these numbers actually mean?

The heritability of a continuous trait is defined as the fraction of the trait’s variance that is due to genetic variation [6]. Mathematically, it is defined as *H*^2^ = *Var*(*G*)/*Var*(*P*) where *Var*(*P*) is the overall trait variance of the trait and *Var*(*G*) is the variance of the genetic component of the trait. While this definition appears simple, it involves certain assumptions and nuances that are easy to miss or misinterpret. First, this definition does not naturally apply to binary traits. To define the heritability of a binary trait (e.g., schizophrenia), it is typically assumed that there exists a continuous liability score underlying its manifestation (i.e., the trait manifests when the liability score is above a certain threshold). The heritability of the binary trait is then defined as the heritability of that latent continuous score [70]. Another important nuance is that heritability is (by definition) context-specific, and not a fundamental property of nature; it is defined for a specific population in a specific time and place. For example, as societies become wealthier, human height becomes less contingent on access to nutrition and, as a result, it is expected that more of the variance would be the result of genetic differences. As a result, we expect to see human height becoming more heritable. Another crucial question about this definition (which we address later) is what exactly is meant by “the genetic component of the trait” (*G*).

There are many motivations for estimating the heritability of a trait. Primarily, it is very useful for guiding genetic research. For example, it could be used to anticipate the theoretical limit of the performance of PRS, letting us know how far we are from a complete analytical model of a trait’s genetic architecture. Likewise, heritability estimates often provide justification for further genetic studies. From a clinical perspective, it can inform family members of their risk to develop a disease diagnosed in their relatives. Heritability sometimes also arises in the face of social questions. For example, there is an ongoing debate in economy to what extent income inequality and social mobility are determined by socioeconomic status at birth vs. immutable genetic factors (as well as indirect effects from the genetics of the parents) [71]. Another motivation for heritability estimates is simple curiosity: studies proclaiming to what extent variations in different traits are due to genetics often attract the public’s attention.

### Methods for estimating heritability

**Fig. 3:**
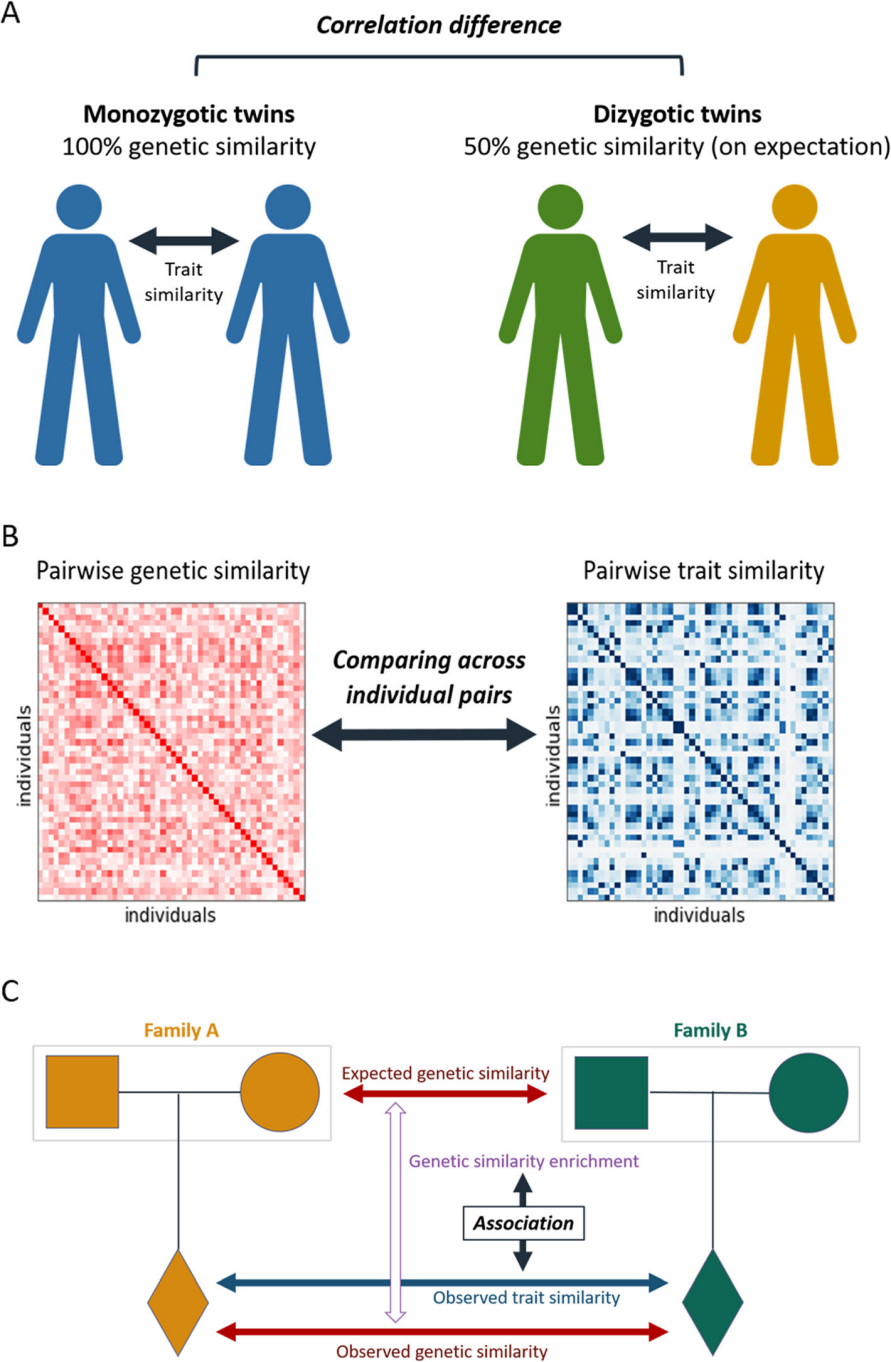
Estimating heritability. Common methods for estimating the heritability of human traits. **A** In twin studies, heritability is estimated by the degree to which monozygotic (identical) twins are more phenotypically similar to each other than dizygotic (non-identical) twins. **B** In GREML, heritability is estimated by comparing genetic and phenotypic similarities across pairs of unrelated individuals. **C** In family-based methods, given a pair of individuals and their parents, the degree to which they are more genetically similar than would be expected from their parents can be compared to their phenotypic similarity to estimate the heritability of the trait

As alluded to, explicitly modeling the genetic component of a trait through PRS generally provides only a weak signal. Therefore, estimating the variance of the genetic component of a trait requires more sophisticated methods. There are many heritability estimation methods. While they often involve complex mathematical analysis, the underlying principle shared by all methods is straightforward: the more heritable a trait is, the more we expect individuals who are genetically similar to also be phenotypically similar. By making certain assumptions about the genetic architecture of the trait and analyzing the association between genetic and phenotypic similarities, a trait’s heritability can be estimated. There are three main categories of heritability estimation methods: (i) twin studies, (ii) genomic relatedness, and (iii) family-based methods (Fig. 3). Each methodological category is suited for distinct types of data, is subject to different assumptions, and has unique strengths and weaknesses.

The classic method for estimating heritability is through twin studies (Fig. 3A) [72]. In such studies, monozygotic (identical) twins are compared to dizygotic (non-identical) twins. If a trait is heritable, we expect monozygotic twins to be more phenotypically similar than dizygotic twins. Knowing that monozygotic twins are 100% genetically similar, whereas dizygotic twins have only 50% chance of having the same parental allele transmitted to them (in variants that are heterozygous in one of the parents), Falconer’s formula can be derived. The formula states that the heritability of the trait is *H*^2^ = 2(*r*_*MZ*_ − *r*_*DZ*_), where *r*_*MZ*_ and *r*_*DZ*_ are the phenotypic correlations between monozygotic and dizygotic twins, respectively [73]. Notably, twin studies make the assumption that monozygotic twins do not share a more similar environment than dizygotic twins. It also assumes lack of genetic (GxG) and gene-environmental (GxE) interactions (which we discuss later). The greatest strength of this method is that, unlike all other methods, twin studies do not require genetic data at all, only phenotypic measurements. For this reason, twin studies had provided heritability estimates long before genetic sequencing became a viable technology.

A second class of methods for estimating heritability, which has become popular since the era of GWAS, leverages genetic cohorts of unrelated individuals and includes methods such as GREML (Genomic Relatedness Restricted Maximum Likelihood) and LDSC (Linkage Disequilibrium Score Regression) [74]. GREML estimates heritability by calculating and comparing all pairwise similarities between a cohort’s individuals with respect to both genotype and phenotype (Fig. 3B). LDSC estimates heritability by relating the estimated effect sizes of variants to their LD scores (defined as the sum of a variant’s squared correlations with all other variants). The more heritable a trait is and the more a variant is in LD with other variants (and thus able to “borrow” signal from them), the stronger effect sizes become (on expectation). LDSC has the advantage that it requires only publicly available summary statistics. An important weakness of these methods is their sensitivity to population structure and other environmental biases [17]. As in GWAS, population structure is commonly accounted for in GREML and LDSC by using the top principal components of genetic variation. However, there are concerning indications that residual population structure could still bias their results (open problem #1: population structure) [10, 17].

A third class of methods utilizes family-based data on genotyped relatives [75]. The key idea here is to assess whether related individuals with greater than expected genetic similarity also share a greater than expected phenotypic similarity (Fig. 3C). By accounting for the genetics of parents, family-based methods are insensitive to most environmental biases undermining twin and cohort studies, including population structure. The family-based approach also captures only direct genetic effects (unlike cohort-based methods which also capture indirect effects) [18]. On the downside, it is harder to obtain a sufficient number of samples required for accurate heritability estimates (due to the difficulty of recruiting families).

### Types of heritability

A crucial difference between heritability estimation methods is the type of heritability they measure. Earlier we gave the definition of *H*^2^, known as the broad-sense heritability. This is the type of heritability that twin studies presume to measure (although, as we later discuss, genetic interactions and environmental biases can lead to overestimation of the true heritability). Another type of heritability, known as the narrow-sense heritability (denoted *h*^2^), reflects only the additive part of heritability, namely the part that can be expressed as a linear combination of individual variant effects. Since the genetic component of a trait could also include non-additive genetic effects, it generally holds that *h*^2^ < *H*^2^.

Heritability estimation methods that rely on genotype data typically capture only the additive genetic effects of the genotyped variants. This type of heritability is called the SNP heritability (denoted ${h}_{SNP}^2$), and it of course depends on the exact set of variants used to estimate it. The question which variants are reflected by a heritability measure (all variants or only a subset) is in principle orthogonal to the question what types of effects are reflected (only additive or also non-additive). But in practice, commonly used methods either presume to measure the entire heritability (*H*^2^), as in the case of twin studies, or only the additive component reflected by genotyped variants (${h}_{SNP}^2$), as in the case of GREML and LDSC.

### Interpreting heritability

In the presence of so many methods for estimating heritability, each defining heritability somewhat differently and making specific assumptions about the genetic architecture of human traits, there is an ongoing debate on which methods should be considered the most reliable and how to interpret the numbers they produce (open problem #10: heritability estimate interpretation). The problem is aggravated by the sensitivity of the methods to modeling assumptions and the fact that different methods often provide different estimates [75, 76], even for methods of the same family [77, 78]. For example, estimates of heritability may range from 45 to 80% for height, 23 to 57% for total cholesterol levels, and 17 to 43% for educational attainment (i.e., number of years of education), depending on the method and population studied (and whether broad-sense or narrow-sense heritability is sought) [75].

As mentioned, one of the motivations to estimate heritability is getting a sense of how much predictive power we can expect from PRS. But does it mean that a trait’s heritability is an absolute upper bound on the performance of a PRS? Since heritability estimation methods are never completely assumption-free, it would probably be a mistake to interpret these numbers as strict upper bounds that could never be exceeded. Furthermore, population structure and indirect genetic effects may allow genetics to capture phenotypic variance above *H*^2^. Rather than a strict upper bound, it is probably more sensible to see heritability estimates as a crude assessment for the theoretical limit of PRS, given our current knowledge.

### The missing heritability problem

It was noticed early on that discovered genetic associations tended to explain only a small fraction of the heritability of most complex traits. The phenomenon was named “the missing heritability problem,” expressing confusion about where most of the heritability was hiding [16]. Later, the focus of the problem has somewhat shifted, and nowadays the term “missing heritability” more commonly refers to the gap between heritability estimates from genotype and twin data [17]. Formally, it is generally the case that ${h}_{PRS}^2\ll {h}_{SNP}^2\ll {h}_{twin}^2$, where ${h}_{SNP}^2$ and ${h}_{twin}^2$ are the heritability estimates obtained from genotype- and twin-based methods, respectively, and ${h}_{PRS}^2$ is the fraction of the phenotypic variance explained by a concrete PRS. Both of these inequalities reflect possible gaps in our understanding of the genetics of human traits (open problem #11: missing heritability). The first gap (${h}_{PRS}^2\ll {h}_{SNP}^2$) is more easily explained by simple statistical considerations, specifically the fact that many complex traits are highly polygenic and involve mostly weak effects (or strong but rare effects), meaning we might not have sufficient statistical power to capture the entire SNP heritability with GWAS and PRS (although this may change with greater sample sizes). The second gap (${h}_{SNP}^2\ll {h}_{twin}^2$) necessarily reflects a failure of at least one of the two heritability estimation approaches to capture the true (broad-sense) heritability *H*^2^.

Some explanations for the ${h}_{SNP}^2\ll {h}_{twin}^2$ gap argue for overestimation of the true heritability by twin studies, due to genetic interactions creating “phantom heritability” or mistaking of the effects of greater environmental similarities between monozygotic twins for genetic effects [17, 76]. Other theories argue for underestimation by SNP heritability. The most prominent of these theories sees rare, ungenotyped variants as the main source of missing heritability. Other explanations point out that SNP heritability could also be biased due to residual population structure [10, 17], environmental biases [75], indirect genetic effects [18], and genetic interactions (discussed later).

Some go as far as arguing that the whole notions of heritability and missing heritability are ill-posed and that these statistical models are based on too many assumptions to be taken at face value [79]. Under this view, it could be more productive to forsake heritability estimates at this point (or at least to treat them as qualitative rather than quantitative assessments) and focus instead on improving predictions.

### Rare variants

Perhaps the most discussed potential source of missing heritability is rare variants. Variants exerting strong phenotypic effects are expected to be under intense selective pressure, and therefore remain at low frequency. As a result, genetic effects on complex traits are usually constrained to either common variants exerting weak effects or rare variants with strong effects; both are statistically hard to detect and quantify.

After analyzing very large cohorts of whole-genome sequencing (which have only become available in recent years), some argue that most of the heritability can now be explained and that the missing heritability problem should be considered resolved [80], but this is still highly controversial [17] and some works argue that rare variants have overall limited contribution to heritability [81]. In mice crossed from two inbred strains, where the allele frequency of all variants becomes either 0/100% or close to 50% regardless of how rare they were in the original population (thereby breaking the natural negative correlation between allele frequency and effect size), genetic associations were shown to explain a much larger fraction of the phenotypic variance, suggesting that the overall contribution of rare variants might be substantial [82]. Whether or not rare variants alone are the main source of missing heritability, by now there is a lot of evidence that they play an important role in many complex traits [83]. Unfortunately, genetic studies still rarely deal with rare variants, and there is shortage of whole-genome and whole-exome sequencing cohorts that can capture them (open problem #4: rare variants).

A key problem in dealing with rare variants is that sequencing is still considerably more expensive than SNP-array genotyping, and reduction in sequencing costs has stagnated in recent years [26]. As a result, there is a real trade-off between the quality and quantity of genetic data, and it is not clear which of the two is more critical. The recent release of hundreds of thousands of whole-exome and whole-genome sequences by the UK Biobank [84], in addition to SNP-array genotypes over the same individuals, could provide valuable insight into the cost-effectiveness of different types of genetic data. Another problem is that even after a large cohort is fully sequenced, it is not entirely clear how to interpret rare variants. Association studies and PRS typically require variants to be frequent enough to accurately assess their effects. Dealing with rare variants may require more sophisticated methods that look beyond the statistical patterns of specific variants. A promising class of methods is burden tests, which consider the aggregated effects of multiple variants sharing the same gene or genomic locus [41–45, 83]. Another approach to analyzing the effects of rare variants would be to leverage family studies [27]. Additional problems with rare variants are that they are more sensitive to residual population structure [17] and quality control (as it is harder to distinguish true variants from sequencing errors in the presence of limited data).

## Non-additive genetic effects: oversight or non-issue?

### The additive genetic model

Nearly all methods for studying the genetics of complex human traits, from GWAS to PRS to heritability estimates, assume additivity in the effects of variants. The typical additive model for a continuous trait is *y* = *β*_1_*g*_1_ + … + *β*_*k*_*g*_*k*_ + *ϵ*, where *y* is an individual’s phenotype value, *g*_1_, …, *g*_*k*_ are the individual’s genotype values over *k* variants, *β*_1_, …, *β*_*k*_ are constant per-variant weights indicating the effect size of each of the *k* variants, and *ϵ* is Gaussian noise (typically assumed to be independent across individuals) capturing the remainder of the phenotype (including environmental, non-additive and random effects, as well as the genetic effects of unincluded variants). When dealing with a binary or categorical trait (e.g., a disease diagnosis), the model is typically altered into *P*(*y* = 1) = *σ*(*β*_1_*g*_1_ + … + *β*_*k*_*g*_*k*_), where *σ*(*x*) =  *exp* (*x*)/(*exp*(*x*) + 1) is the standard logistic function, and *P*(*y* = 1) is the probability of the individual to have the trait. It is also common to include in the model other covariates in addition to the genotype values, such as sex, age, and the principal components of genetic variation (to account for population structure). Consistent with the overall assumption of additivity, the genotype values *g*_1_, …, *g*_*k*_ are typically also encoded in an additive way, namely each *g*_*i*_ is set as 0, 1, or 2, depending on the number of copies of the alternative allele of variant *i* that the individual has. Following the 0/1/2 encoding, genotype values are commonly normalized such that each *g*_*i*_ would have a mean of 0 and standard deviation of 1 over the study cohort.

Within this framework, *y* and *g*_1_, …, *g*_*k*_ are observed, and the study’s goal is to make inference about the coefficients *β*_1_, …, *β*_*k*_. In GWAS, one is typically interested in finding out whether *β*_*i*_ ≠ 0 for each of the tested variants by calculating a *p*-value for the null hypothesis *H*_0_ : *β*_*i*_ = 0. In PRS, the goal is to simultaneously estimate all of the coefficients *β*_1_, …, *β*_*k*_ and to use the expression *β*_1_*g*_1_ + … + *β*_*k*_*g*_*k*_ as a predictor for the phenotype *y*. In heritability estimation, the objective is to estimate the overall genetic variance *Var*(*β*_1_*g*_1_ + … + *β*_*k*_*g*_*k*_).

### Is the additive model (more or less) true?

It is obvious to anyone familiar with complex biological systems that genetic effects are not truly linear. Nonetheless, many argue that linear genetic models are a good-enough approximation of the real biological complexity [85]. According to this common view, the contribution of non-additive genetic effects to the variance of most traits is low (i.e., additive genetic effects account for most of the heritability), so we need not worry too much about them. In other words, there might be a stark contrast between the prevalence of biological epistasis (variants interacting at the molecular level) and the overall magnitude of statistical epistasis (variants whose collective contribution to the phenotype at the population level deviates from a linear model) [86].

By now, the additive genetic model has become so mainstream that it is commonly just taken for granted without any explicit justification. Despite its popularity and despite being very convenient for computational and statistical analysis, it is important to understand the empirical and theoretical support in favor of the additive genetic model, and consider the possibility that it might nonetheless be wrong or incomplete.

A strong empirical result in favor of the additive genetic model was presented in a 2015 meta-analysis covering virtually all twin studies published between 1958 and 2012 [72]. It was found that, across many different traits, the phenotypic correlation between monozygotic twins was roughly twice the correlation between dizygotic twins, consistent with a model of mostly additive genetic effect and no shared environmental influences on twins. Specifically, 69% of the 2,748 analyzed twin studies were consistent with the null hypothesis *r*_*MZ*_ = 2*r*_*DZ*_. However, results in this meta-analysis were shown only for groups of traits (such as “cognitive traits”), but not for individual phenotypes. Furthermore, the heritability of some traits (such as height) had been studied much more extensively than others, and most of the analyzed studies had investigated continuous (rather than binary or categorical) traits. Another limitation acknowledged by the authors was the presence of substantial overlap in the twins recruited across the different studies.

Also supporting the additive model is the poor track record of nonlinear phenotype prediction models, which generally do not substantially outperform linear PRS. On the other hand, there have not been that many attempts to develop such models, and it might be too early to give up on outperforming linear PRS (see next section).

Another argument in favor of additivity appeals to theoretical considerations that, under certain assumptions and in the presence of a sufficiently large number of causal variants (i.e., if the trait is sufficiently polygenic), the overall phenotypic variance attributed to additive genetic effects will almost certainly dominate the epistatic (i.e., non-additive) genetic effects [87]. On the other hand, a recent simulation analysis of phenotypes with deep neural networks showed that phenotypes that cannot be approximated well by a linear model are possible [88].

Empirical evidence against the additive model includes the fact that epistatic genetic effects are often detected when sought, and possibly contribute to phenotypic variance quite substantially in some non-human organisms (see later section). Another argument opposing the additive genetic model is that we still have fundamental gaps in our understanding of the genetic architectures of complex human traits, and the assumption of additivity (made by virtually all heritability estimation methods) should be considered an immediate suspect for the missing heritability problem [76, 79]. Given what we know about complex biological systems, the burden of proof should be on those arguing for additive genetic effects being the primary source of heritability.

It is also important to note that a lot of the research and discussion on the genetic architecture of complex human traits, arguably too much of it, has focused on a rather small set of human traits, and especially on human height. It is possible that we have been somewhat led astray by focusing too much on a non-representative example. Height is actually rather unusual within the human phenotypic landscape in how heritable it is, and it is also very polygenic (although there are more polygenic traits [34]). Perhaps it is also uncommonly additive. It is also possible that continuous traits, whose genetic architecture is easier to study, behave differently than diseases and other binary traits.

Despite many years of debate on the question, we argue that the question of additivity is still not fully settled and that more work is needed to determine whether non-additive genetic effects underlie substantial phenotypic variance in complex human traits (open problem #2: non-additive and epistatic genetic effects). It would also benefit the discussion on additivity if the claims being defended or argued against were more precisely defined. For example, there is a huge difference between arguing that over 50% of the phenotypic variance attributed to genetics could be approximated by a linear model, which is a rather modest claim, and arguing for near 100%, which is a much stronger claim. We would also like to see evidence in favor or against genetic additivity being presented over more diverse human phenotypes and with more clarity and rigor, in particular with respect to the modeling assumptions underlying such works. When reporting or interpreting the results of genetic studies, it is important to be mindful of the assumption of additivity.

### Consequences of non-additivity to heritability and PRS

The question of additivity is tightly related to the question of missing heritability (open problem #11: missing heritability) and is therefore also relevant to the performance of PRS (open problem #14: genotype-to-phenotype prediction performance). As virtually all contemporary heritability estimation methods assume additivity, non-additive genetic effects could be part of the explanation for the missing heritability problem. As mentioned, SNP heritability does not include non-additive variance, so it is expected that methods such as GREML and LDSC would underestimate the full (broad-sense) heritability. It was demonstrated through simulation analysis that methods for SNP heritability estimation may dramatically underestimate the heritability of phenotypes with nonlinear genetic effects (and that the more nonlinear the simulated effects are, the less heritability is recovered) [88]. Twin studies, on the other hand, assume that the correlation between the genetic effects of non-identical twins is exactly half, which holds for additive but not epistatic effects. As a result, twin studies are likely to overestimate the true heritability in the presence of epistasis [76]. Part of the gap between ${h}_{SNP}^2$ and ${h}_{twin}^2$ could therefore be attributed, at least in principle, to non-additivity.

If non-additive effects indeed make up a big part of the heritability of complex traits, then this has important ramifications for PRS: we would expect linear models to be limited, at least in principle, compared to the full potential of nonlinear models. Some attempts have been made to train PRS with nonlinear models, including support vector machines, random forests, and deep neural networks [89–91]. These nonlinear algorithms have a strong track record across numerous domains of machine learning. However, in the context of PRS, such attempts have generally failed to outperform simple linear models, providing strong evidence in favor of the theory stating that nonlinear effects are not that important in the grand scheme of things, but these indications are not yet decisive. Attempts to model genetic effects in a nonlinear way have only been made a handful of times, and there could be other reasons for off-the-shelf machine-learning algorithms having a hard time to pick nonlinear genetic effects. For example, we know that in genetics, to a much greater extent than in other domains where machine learning is commonly applied, data is often very noisy, effects are very weak (or rare), and there does not exist an obvious and easily-exploitable structure to the data. It would be interesting to see if future works are able to capture a substantial non-additive genetic signal.

The adequate performance of linear models compared to nonlinear alternatives is the primary reason for their dominance in genetic studies, but there are also other motivations swaying researchers to favor them. Other advantages of linear models include high robustness and interpretability, both are crucial for clinical applicability. Also of great importance to researchers is the capacity to work with summary statistics of published results without being dependent on individual-level data. This is easily done with linear models, but usually impossible with nonlinear algorithms. Since human genetic datasets with individual-level information tend to be highly restricted (due to privacy concerns), this is often a critical consideration. If large-scale, highly accessible biobanks (such as the UK Biobank) become more prevailing in the coming years, we might see nonlinear methods becoming more popular.

### Epistatic, dominant, and recessive effects in complex traits

Related to the principal question of non-additivity is the more practical problem of finding epistatic genetic interactions (known as GxG), namely finding combinations (usually pairs) of variants or genes that interact together in a nonlinear way (biologically or statistically) [92]. From a statistical perspective, this is a notoriously difficult combinatorial and computational problem [4]. For example, scanning a dataset with a million genotyped variants for all possible pairwise interactions would involve half a trillion (~5E11) variant pairs. Obtaining sufficient statistical power to find significant pairs under such conditions would require much stronger effects than those required to find additive genetic effects. Even if additive models are in fact capable of capturing most of the genetic component of phenotypic variance, finding epistatic effects might still be important for understanding the mechanism of human traits.

A lot of epistasis research has focused on non-human model organisms, where the phenomenon could be studied with experiments (including genome screening projects with libraries of double gene deletions) [4]. Specifically, a lot of research has revolved around yeast. The general conclusion from these studies is that epistasis is indeed quite prevalent in non-human organisms and could contribute quite substantially to the phenotypic variance [93, 94]. However, many of the experimental studies involve artificially induced mutations or inbred populations and therefore do not provide direct evidence on the scale of the phenomenon with respect to natural genetic variation.

Recessive inheritance is an interesting special case of non-additive genetic interactions. While dominant and recessive inheritance play an important role in the study of Mendelian traits, these inheritance modes are hardly studied in the context of complex traits. In principle, dominant and recessive genetic effects are perfectly applicable to complex traits as well as Mendelian. It should be noted that the textbook definitions of dominant and recessive effects are often just approximations of the genetic effects found in the real world. For example, there are Mendelian diseases exhibiting imperfect recessive inheritance, as in the case of thalassemia (a type of anemia caused by mutations in the hemoglobin genes), which is considered a recessive disorder, but individuals who carry one copy of a deleterious mutation may develop mild symptoms of anemia. Since strong genetic effects are mostly confined to rare variants, an additive model is usually a good approximation for dominant effects but a very poor approximation for recessive effects. The additive model mentioned earlier can in principle accommodate dominant and recessive effects at the variant level through changing of the encoding of the genotype values *g*_*i*_. Specifically, assigning the values 0/0/1 or 0/1/1 instead of 0/1/2 would capture recessive or dominant effects at the variant level, respectively. However, it is anticipated that many recessive effects would occur at the gene level and not at the variant level. If the two copies of a gene are affected by different variants, a case known as compound heterozygosity, then a variant-level recessive model would be completely blind to it. Due to the inherent blindness of GWAS to compound heterozygosity, the study of recessive genetic effects in complex human traits is highly neglected, but some recent works show that they are in fact common in complex traits [95, 96]. Gene-based methods (as opposed to variant-based methods) seem especially promising for detecting recessive effects [45].

## Acknowledging the complexity of genetics

### Gene-environment interactions

Similar to epistatic effects (GxG), interactions between genetic and environmental factors, known as GxE, also play a role in complex human traits [12, 97]. GxE interactions indicate that the effect of a genetic factor is dependent on the presence of an environmental factor (or, equivalently, that the effect of the environmental factor is dependent on the genetic factor), where “environmental factor” means any non-genetic determinant of the trait (including epigenetics). An obvious example is the effect of sunlight exposure (an environmental factor) on melanoma (a phenotype) being dependent on the genetic variants that determine skin color. A less obvious example is different levels of colorectal cancer and adenoma risks associated with the missense variant A222V in the MTHFR gene (methylenetetrahydrofolate reductase) depending on the levels of folic acid dietary intake [97]. Another compelling example of GxE is seen across many autoimmune diseases where it is believed that, in the presence of genetic predisposition, the disease might be triggered by an infection from specific viruses, bacteria, or other pathogens. For example, specific oral bacteria are associated with rheumatoid arthritis [98], and Epstein-Barr virus has been associated with multiple sclerosis [99].

There are many pragmatic reasons to be interested in GxE. By taking into account the modifying effects of environmental factors, more genetic associations could be found [12]. Furthermore, gene-environment interactions can provide insight into the mechanism of genetic associations (open problem #13: from causality to mechanism). Additionally, when GxG or GxE interactions exist, it is possible that genetic associations and PRS will not perform uniformly across human groups (and across space and time). Specifically, it might be possible to identify subgroups showing unusually high risk for genetic or environmental factors. As a result, GxE interactions could allow individualized genetic-based interventions by modifying the interacting environmental factors (thereby reducing disease risk) [12]. Special interest in GxE is present in the social sciences (especially in psychology), where gene-environment interactions are believed to play a central role in human personality and behavior, and an active debate is still going around the old question of “nature versus nurture.”

While generally recognized an important piece in the genetic puzzle of complex human traits, GxE is a notoriously difficult subject of study, and there has been limited progress in addressing it (open problem #3: gene-environment interactions) [100]. It is very challenging to run reliable and robust GxE studies. For example, it is difficult to properly address potential confounding, to an even larger extent than in standard genetic studies [100]. Over the years, there have been few concrete GxE discoveries, and even fewer successful replications. Discovering GxE interactions is hard in part due to the vast combinatorial search space (as in GxG) [97]. When the genetic variants and environmental conditions under investigation are both rare, detecting an interaction between them becomes nearly impossible. On top of all the regular challenges in replicating genetic associations, GxE replication studies and meta-analyses are often hindered by differences in how environmental factors are defined and measured across studies [12]. Even rigorously defining what exactly GxE means is not trivial, as it requires to describe what the architecture of a trait should look like in the absence of interactions (when both genetics and the environment may influence the trait, but separately). Specifically, the presence or absence of GxE could depend on the scaling of the phenotype. In the case of a binary phenotypic outcome and binary genetic and environmental factors, it would depend on whether we expect risk for the outcome to depend additively or multiplicatively on the separate risks associated with the genetic and environmental factors [97]. When the interacting environmental factors are themselves affected by genetic factors, GxE interactions could be mediators of underlying GxG interactions, meaning that GxG and GxE are often tightly related.

Various statistical methods and computational approaches have been developed to detect GxE interactions [100]. In some cases, the study of GxE interactions can be informed by cellular experiments, if the organism-level exposures can be translated into cellular exposures that can be tested in the lab (e.g., cytokines as a proxy for inflammation).

The study of GxE has important ramifications for the concept of heritability and the question of missing heritability. There have been works trying to quantify the overall contribution of GxE to phenotypic variance (for certain collections of environmental factors, and under certain assumptions about the genetic architecture of the studied traits). These attempts have often ended up with the conclusion that GxE interactions contribute quite substantially to the phenotypic variance of some traits (such as BMI and pulse pressure) [101, 102], yet the overall importance of GxE to trait heritability is still controversial [85, 100]. Notably, the mere definition of heritability is tricky in the presence of GxE interactions. To define heritability, continuous phenotypes are commonly decomposed as *P* = *G* + *E*, where *P* is the phenotype value, *G* is the overall contribution of genetic factors to the phenotype, and *E* is the contribution of environmental factors (defined as the residual term encapsulating all non-genetic factors contributing to the trait). It then holds that *Var*(*P*) = *Var*(*G*) + *Var*(*E*) + 2*Cov*(*G*, *E*). This means that when *Cov*(*G*, *E*) ≠ 0 (in the presence of GxE interactions), *Var*(*G*) will not capture all the variance of the trait that is due to genetics, as implicitly assumed by defining heritability as *H*^2^ = *Var*(*G*)/*Var*(*P*) (and if *Cov*(*G*, *E*) is negative, it may even capture too much). It has been argued that environmental effects, when not properly accounted for, may indeed inflate heritability estimates. For example, it has been asserted that heritability estimates for educational attainment might be inflated by ~70% [75]. Some have gone as far as arguing that just as we cannot say how much of the area of a rectangle is due, separately, to each of its two dimensions, so it is not possible to separate “nature” from “nurture” [79].

### Selection bias

Selection bias describes a situation where a study cohort does not perfectly represent the population that it is presumed to represent, leading to unjustified conclusions about the general population. Since participation in human studies is generally voluntary, this is a difficult challenge to overcome. While the problem and the challenges it presents are by no means unique to genetic studies, human genetics researchers appear to have been surprisingly unconcerned about the implications of selection bias. Only very recently did the problem gain some attention, following a few demonstrations of its potential to lead to false discoveries. For example, a recent study detected statistically significant associations between autosomal variants and sex [103]. Since such associations are clearly spurious (autosomal chromosomes cannot affect sex), it was concluded to be the result of participation bias. Specifically, if both sex and a particular genetic variant affect an individual’s decision to participate in the study cohort (which can be seen as a behavioral trait), then we would have a collider bias when conditioning on that decision. Another special case of selection bias is survival bias. If we recruit individuals with a history of cancer to participate in a genetic study, then it could be biased by individuals with extremely aggressive tumors not having survived to participate. It was also demonstrated that the common case-control study design (where cases and controls are recruited separately) can lead to underestimation of SNP heritability, thereby adding to the missing heritability [104]. Since the problem of selection bias in genetic studies was acknowledged only recently, not enough work has been dedicated to fully understanding the scope of the problem and working out methods and best practices to minimize its effect (open problem #9: selection bias).

### Overlooked genetic variation

We have deliberately focused this review on problems that are specific to the link between genotype and phenotype, putting aside most of the upstream technical aspects of collecting genetic data (such as issues related to sequencing, variant calling and quality control). Nonetheless, it is crucial to acknowledge that, like in any field, the results of our research will only be as good as the quality of the data we use. In particular, there are certain types of genetic variation that are systematically underrepresented, or altogether absent, from contemporary genetic studies (open problem #5: non-standard genetic variation).

We have already touched on the challenges of studying the effects of rare variants due to data scarcity (open problem #4: rare variants). Other neglected variants are those in sex chromosomes (X and Y) [95], which are often excluded from genetic studies due to mapping and variant calling challenges, and also because they diverge from diploidy and, as a result, from common modeling assumptions. The mitochondrial chromosome (MT) presents similar challenges and is even more commonly left out. Structural and copy number variations also pose a major challenge to existing protocols and tend to be ignored, as they are hard to genotype and sometimes also deviate from standard modeling [16, 105, 106]. For example, it is open to interpretation what dominant or recessive inheritance would mean in the case of copy number variation. Genetic variation in repetitive regions of the genome also tends to be ignored due to mapping challenges [107]. Part of the gap between heritability estimates from twin and genotype data could be due to under-genotyped genetic variation [16, 17, 79]. Advanced genotyping technologies such as nanopore and PacBio sequencing may play a role in resolving complex genetic variants [108, 109].

Another aspect of genetic variation that tends to be overlooked is phasing, namely whether two heterozygous variants occur on the same or different copies of the chromosome (and their maternal or paternal origin) [110]. This could be important, for example, when dealing with compound heterozygosity (where a recessive effect takes place only when variants occur at both copies of a gene) or epistatic effects involving cis-regulatory elements. Another aspect of human genetic variation regularly neglected is mosaicism, namely the occasional occurrence of mutational events during cell division leading to genetic diversity between cells in the body (with more cells having a genetic alteration the earlier it occurred during development) [111]. Genetic studies assume that an individual’s genetic sample represents their entire genetic repertoire, but that is not perfectly accurate. There is evidence suggesting that genetic mosaics could have phenotypic effects in the brain in traits such as autism spectrum disorder [111]. Whether this is an anecdotal phenomenon or an important aspect of human genetics still remains to be determined. Should mosaicism be taken into account in genetic studies, or is it better treated as yet another non-genetic factor (like other omics and environmental effects)?

A related question is how to represent genetic variation. The accepted norm today is to represent an individual’s genome as a set of unrelated variants indicating deviation from the human reference genome (which is itself a somewhat artificial and incomplete construct [112]). Is it necessarily the best way to represent genetic variation and study its effects on human traits? A recently explored alternative is to skip variant calling altogether and to seek direct associations of the trait with raw sequencing reads [113]. Another long-standing idea is to represent genomes as graphs of haplotypes [114].

In a sense, we have only been dealing with the easiest parts of human genetics, focusing on common, simple autosomal variants, without genetic and environmental interactions. The overall contribution of unaccounted genetic variation to heritability, and whether this is a major source of missing heritability, is yet to be determined.

### Phenotype definition

Just as the quality and representation of genotypes is expected to have a major influence on the results of genetic studies, so can the exact definitions and measurements of human phenotypes be important. While some phenotypes are straightforward to measure accurately (e.g., height), other traits are a lot more nuanced and could be defined in different ways. For example, there has been a long discussion on whether schizophrenia and bipolar disorder, two distinct but highly related psychiatric diagnoses with strongly overlapping genetic determinants, should be seen as residing on a single psychosis continuum [115]. Moreover, different physicians may end up assigning the same individual with different diagnoses [116], and some traits are prone to diagnostic errors [117]. Diagnostic protocols may also vary between health or insurance systems, even within the same country. A related question is whether to define disease status based on official clinical diagnoses or self-reports [59]. Ill-defined phenotypes are expected to introduce random noise and systematic biases into genetic studies [118].

It has been postulated that some clinical diagnoses are in fact a combination of distinct biological phenotypes, each involving separate genetic and cellular pathways, and that the similar clinical manifestations of these different phenotypes are only superficial. In such cases, it could be beneficial to study each of the subphenotypes separately [15]. Conversely, it is also possible that some diagnoses that are considered distinct in fact share a common biological etiology and are therefore better defined as a single phenotype. There are some preliminary efforts to use genetics as a guideline for better phenotype definitions [119]. Improving the methodology and practices used to define and measure phenotypes would be highly beneficial (open problem #8: phenotype definition).

Sex differences, which are prevalent across the human phenome, could be seen as an important special case of subphenotypes [15]. When sex interacts with genetics in a non-additive way, sex differences can also be seen as a special case of GxE and GxG interactions (in this case, the interaction of sex chromosomes with other genetic factors). The common practice today in GWAS and other genetic studies is to treat sex as yet another covariate that has to be accounted for. But given its profound effect on so many human traits, perhaps more comprehensive approaches are needed. For example, it might be a constructive norm to study and report on males and females separately, when sample sizes and statistical power allow that.

## Open discussion on open problems

We have attempted to provide a broad view on current important open problems in the field of human trait genetics. Towards this goal, we reached out and spoke with many active researchers in the field covering a diverse set of backgrounds and perspectives. Following these conversations, we converged on a list of 16 open problems that were consistently acknowledged by us and others to be particularly important (Table 1). Our main criterion for deeming an open problem sufficiently important was: would it considerably help realizing the field’s potential if this problem were solved?

We attempted to provide an up-to-date view on the field, focusing on the open problems that appear to constrain the field in the near future. Despite the efforts we have made, we are aware that this review and our list of open problems are far from being truly exhaustive. There are many additional relevant open problems that we judged to be relatively minor, and it is also very plausible that we have overlooked some major open problems.

We hope that this presentation of open problems will contribute to the open discussion already taking place. In particular, critical discussion on this list of open problems (and on open problems we have overlooked) would be beneficial. We think that an open dialog is especially important among researchers and practitioners from diverse backgrounds and expertise, for example between researchers and clinicians. Other important discussions should include technology providers (e.g., of high-throughput sequencing and data processing) and resource providers (such as biobanks). For example, whether data resources are dominated by publicly available biobanks or restricted databases will greatly affect the kind of challenges we face.

## Supplementary Information

**Additional file 1.**
