# Supplementary material for: Open problems in human trait genetics
Source: Genome Biol. 2022 Jun 20;23:131. doi: 10.1186/s13059-022-02697-9 (PMC9208223; doi:10.1186/s13059-022-02697-9)
Supplement: Supplementary file 1 — Additional file 1. [file 13059_2022_2697_MOESM1_ESM.docx]

**1^st^ round**

**Reviewer 1**

Brandes et al. have written an interesting and timely perspective on open problems in human genetics. Based on discussions with some colleagues, they have compiled a list of important problems that remain unsolved, either due to lack of data, appropriate methods, or lack of attention. While they make few points that are entirely novel and not observed in any previous literature, compiling the points in this format is valuable and insightful, and is a solid contribution to the literature. I can see this being particularly valuable for trainees that are trying to map out the frontiers of the field. The manuscript is well written and fun to read.

I have some comments - in the order of appearance - that are mostly suggestions of clarifications, some additional points, or references:

I find Table 1 to be too condensed to be helpful or more than a table of contents. I'd suggest making it more like Supplementary Table 1 where the essential points are summarized.

"The required sample size is determined by the allele frequency and effect size of each association." - also by distribution/prevalence of the trait, especially for case-control traits where rare phenotypes require larger sample sizes.

"Focusing on homogenous ethnic groups also underplays the potential genetic basis of phenotypic differences between groups." ..And excludes a large proportion of humans with more complex ancestries

In the Population structure section, the authors use the word "ethnic" (identity/diversity/group) quite a lot. This may be appropriate depending on the exact meaning, but in the American context this has a rather specific use (Hispanic / non-Hispanic) that may confuse things. Also, it should not be used as ~synonymous to ancestry, and sentences such as "Individuals of European ancestry are overwhelmingly overrepresented in contemporary cohorts and, as a result, most published results do not directly transfer to other ethnic groups" sound somewhat problematic to me, contrasting European ancestry and other ethnic groups. The terminology around this issue is terribly confusing and somewhat controversial (see e.g. https://arxiv.org/abs/2106.10041 and https://www.nature.com/articles/s41588-021-00952-6 ) but (thus?) I'd encourage the authors to take another look at this and make sure their terminology is at least consistent and as appropriate as possible. In many places, replacing "ethnic" with "ancestry/ancestries" might be better?

"Like most genetic resources, haplotype reference panels are highly biased towards individuals of European ancestry, and so are the popular DNA microarrays (which usually genotype variants present in these populations). As a result, the genetic coverage obtained for non-Europeans is typically much lower" This is not untrue, but isn't this mostly a legacy issue? TOPMed imputation panels and e.g. the MEGA array are not that bad in terms of diversity. Is the coverage in current studies really MUCH lower for non-Europeans?

"However, gene-based approaches are still susceptible to LD; it is possible that signal from nearby variants would leak into variants affecting the gene." Furthermore, it's not uncommon for expression of nearby genes to be coregulated - this is true biology of joint regulatory elements and would persist even if there was no LD leakage - and when that happens, multiple genes will be implicated even though only one might be causally related to disease. There's ~no way for genetics to disentangle this.

Regarding competitions and challenges for causal variant inference, this might be a good reference: https://onlinelibrary.wiley.com/toc/10981004/2019/40/9 - this has been done to some extent.

"could in fact be considered omnigenic, namely that they are affected by most of the genes in the genome [43]"…that are expressed in the trait-relevant tissue(s)

"In such cases, additional mapping of genomic loci associated with omnigenic traits, without considering other factors (such as effect sizes), is not expected to substantially contribute to our understanding of
mechanism." - this, in the context of the preceeding section makes too strong claims, in my opinion. The omnigenic model is essentially a hypothesis that has received a fair amount of criticism and lacks empirical data that could provide the fundamental support/falsification for its assumptions. Even if true, they way to assign traits as omnigenic or not is not defined. I think some of these uncertainties should be reflected on here.

In the section "From causality of mechanism" the authors basically skip over the substantial amount of mechanisms-focused work that does not rely on perturbation experiments. I acknowledge that this is not the focus of the manuscript, but not mentioning efforts to annotate regulatory elements, causal genes, affected networks and relevant cell types of GWAS traits using data sets like ENCODE, GTEx and HCA is an oversight. This is essentially skipping over 15 years of functional genomics work to describe the next hot thing in town: perturb-seq (which is still largely untested as to its uses in GWAS interpretation).

Also, perturb-seq is a method to link gene knock-outs - not genetic variants - to gene expression. To my knowledge, there is no high-throughput perturbation approach (yet) "to detect variants that causally affect gene expression".

"A useful practical approach to improving PRS performance is to incorporate clinical factors into the
predictive models on top of genetic markers (e.g., using body mass index and birth weight to
improve type-2-diabetes PRS [48])." My understanding is that while PRS alone can often work pretty well, one big practical challenge is that the marginal benefit over already used clinical predictors is modest. This may be worth mentioning in a clearer way.

Transferability of PRS: I strongly recommend adding this reference here and in other applicable places: https://pubmed.ncbi.nlm.nih.gov/31999256/

"but health providers would still want to see good evidence" -> health care providers

I find the heritability section to be interesting and well-written, but the first few pages of, discussing methods to estimate heritability, it reads largely like a review, rather than the topic of this manuscript: defining open problems in genetics. This content could be condensed (or re-used in another context) to highlight the open questions in the area rather than existing methods.

"In inbred mice, where the allele frequency of all variants becomes either 0% or close to 50% regardless of how rare they were in the original population…" I don't get why it would be 50%. Shouldn't it be 0% or 100%, i.e. one or the other allele is fixed in the inbred strains? Am I missing something? F1 crosses would have a lot of 50% variants but not inbred parents.

"The recent release of 200K whole-exome sequences by the UK Biobank…" Now we already have genomes! :-) Please update.

"Given what we know about complex biological systems, the burden of proof should be on those arguing for additive genetic effects being the primary source of heritability." Do you indeed mean heritability, or missing heritability?

In the section about additive versus non-additive genetic effects, it might be worthwhile to mention the distinction between and discussion around statistical versus biological epistasis.

Just a suggestion for the GxE part: autoimmune disease risk is a particularly compelling example of GxE being relevant for fundamental understanding of overall disease mechanisms, with rather compelling hypotheses and data pointing to a disease architecture where genetic variants predispose to inappropriate response to external microbial stimuli, and these jointly cause disease.

Regarding the difficulty of finding GxE, it would be worth mentioning that functional analyses of cell state specific molecular effects of GWAS loci has been quite a powerful way to characterize GxE, even when the GxE effect may be difficult to detect at the GWAS level. In particular, response eQTL studies in immune cells have been important here.

"Just as the quality and representation of genotypes is expected to have a major influence on the results of genetic studies, so do the technicalities of how human phenotypes are defined and measured." - I think this is an issue of more than just technicalities; some of the problems of phenotyping (as discussed in the text) are actually very profound. I'd suggest dropping that word.

**Reviewer 2**

Summary/General comments:
In this manuscript by Brandes, Weissbrod, and Linial, the authors put forward a set of open questions in the field of human trait genetics. These questions span an expansive range of issues that include general issues (e.g. population structure, epistasis, GXE interactions), data-related issues, and specific challenges that persist in the field of human genetics. The authors address ongoing issues, biases, and limitations of genome-wide association studies (GWAS), heritability estimation, and polygenic risk scores with an aim of providing a helpful primer for geneticists, students, and researchers new to the field of human genetics. The authors propose a number of interesting questions and spotlight some areas that remain largely unaddressed - such as the underrepresentation of sex chromosomes in genetic discovery effots and the unexplored consequences of non-additivity in trait mapping and genetic prediction.

The scope of this manuscript is undoubtedly ambitious, and indeed is ripe with questions that are relevant to the field of human genetics and necessitate further exploration. However, there are areas where this manuscript can be substantially improved. The breadth of issues addressed is expansive, and at places seem disjointed which disrupts the narrative flow and readability. Moreover, the depth of content and discussion is lacking in certain subsections. I found some sections to be interesting and edifying (such as the sections on heritability and non-additivity), but others to be superficial ("direct vs indirect effects"). I think that all sections would benefit from additional vignettes that illustrate the problems and proposed solutions in greater detail. Too often, as I was reading through a section, I would be introduced to a problem, become interested in the problem, and then be introduced to a new problem in a different section without being sufficiently convinced or finding resolution in the previous section. The authors do provide citations at these points, but the manuscript would benefit from elaboration of specific examples or vignettes from these cited works, rather than passing references or one sentence descriptions. Moreover, there may be more coherent ways of organising the topics addressed in this manuscript that would elevate the readability and impact of this work. For example, by splitting it into two main sections that track along the two main applications introduced in the Scope section or, alternatively, by introducing broader themes and then having sub-sections corresponding to applications (for example, by introducing non-additivity at a high-level and then have subsections or vignettes that explore the consequences of non-additivity on heritability estimation, GWAS, and PRS). There are many interesting ideas presented in this manuscript and I believe that with further revision this could become a useful resource to the field.


Major comments:
Writing seems needlessly verbose at various points. For example, in the sentence "The notion and tradition of publishing 'open problems' is borrowed from the mathematical disciplines, where the promotion and explicit discussion of major open challenges has a great role..". The words "notion", "promotion", and "great" seem superfluous as they don't substantively advance the argument. I found such long sentences to be a bit distracting and I think the readability of the manuscript would considerably improve with further revision.
On line 55 of Scope (page 5) you indicate that "functional elements in the human genome" is a topic "related to genetics but not to phenotypic variation in present-day humans". Yet, in the previous paragraph you indicate that a primary application is to "obtain insight into biological mechanism at the molecular and cellular level underlying the disease or trait under study". This seems to belie the extensive work based on integrating functional genomic information (e.g. enhancer elements, chromatin accessibility, hypo-methylated regions), which is often tissue- and cell-type specific, at non-coding loci associated with complex traits in order to resolve molecular mechanisms that influence phenotypic variation.
On line 35 of page 7 ("Genetic association studies and their limitations"), population structure and linkage disequilibrium are listed as the "two main issues limiting one from inferring a causal link". These are certainly challenges that can confound association testing and complicate fine-mapping efforts, but I would think that the fact that most GWAS associations are non-coding would also be a main challenge in resolving a "causal link" between a variant and phenotype. More generally, inference of "causality" through association studies is not only complicated by sources of confounding (i.e. population structure) but also from a lack of biological plausibility (e.g. non-coding loci without any corroborating functional evidence). Issues due to bias and chance (e.g. Winner's curse), reverse causality, and inconsistency of associations are also relevant.
The problem of "residual population structure" is certainly interesting and provokes the question of how does one sufficiently control for population structure. How many PCs should one use in their GWAS? 5? 10? 20? It seems there is a lack of consistency in the field here that the authors could elaborate on further. A recent article by Florian Privé (Privé et al. 2020. Bioinformatics) proposes an interesting framework for generating and selecting PCs and applies it to UK Biobank data, indicating that an appropriate number of PCs is highly context specific.
The section on PRS could benefit from further elaboration on how PRS differs considerably from other standard risk factors (e.g. age, BMI, blood chemistry assays, etc.). For example, DNA, unlike other risk factors, does not change throughout one's lifespan (though interaction effects between PRS and other risk factors may differ temporally). It therefore need only be measured once. Moreover, a patient's genotype can be integrated with genetic information from many traits to derive many different PRS scores simultaneously.
In the second paragraph on page 17, it would be interesting to provide a description of LDSC in addition to GREML. Also, the previous paragraphs seem to address broad-sense heritability, whereas these approaches for genomic data pertain to narrow-sense heritability, no? Isn't it important to differentiate between these two forms of heritability and discuss how this affects interpretation of heritability estimates from these approaches?


Minor comments:
Not sure if "robust causal links" can be immediately gleaned from GWAS association results (Scope; page 5; line 38)
On lines 56-58 (page 5, Scope), in what sense would Mendelian Randomization be considered a method that "uses genetics only" to study relationships between traits, but not GWAS?
Page 6, line 8, Scope, it doesn't seem accurate to say that Mendel's first law dictates "pure dominant or recessive inheritance with high penetrance", as the first law (i.e. law of segregation) merely states that two copies of each hereditary factor (i.e. allele) are separated during the production of gametes. The first law does not explicitly address modes of inheritance or penetrance.
Given that two of the three target audiences for this manuscript, as described in the letter to the editor, are early stage students and researchers new to the field of statistical genetics, it may be useful to provide high-level descriptions of PCA (and perhaps LMMs) as they are important for contemporary GWAS but may not be familiar to the uninitiated.
Page 8, fourth paragraph, it seems that family-based trio studies do not directly "account for population structure" but rather obviate the problem altogether. I do find the proposal that incorporating both related and unrelated together in the same study interesting as described in the beginning of page 9. Can you provide an in-text example of how this strategy has been used to improve trait mapping/ GWAS power?
I'd also cite the TOPMED study in addition to the HRC in the section on imputation. Probably worth mentioning that the lack of non-European representation in genetic studies immediately undermines imputation quality of non-European genotyping data.
Page 11, third paragraph, overall this paragraph is vague. Would be better to either elaborate on what the "powerful tools" and questions of interest are, and the specific biases, or simply remove this paragraph.
Seems that the section of direct vs indirect effects may also benefit from a discussion of pleiotropy which can certainly complicate the aim of resolving causal mechanisms between GWAS variants and phenotypes.
According to Boyle et al 2017, the key insight from the "omni-genic" model is not that complex traits are "affected by most of the genes in the genome", but rather that polygenicity can be understood as an interplay between a subset of core genes influencing expansive gene regulatory networks, and many peripheral variant effects on genes outside the set of core genes. Hence, the heritability may involve the effects of many variants (akin to polygenicity), but only a subset of genes will be relevant to causal mechanisms and drug target discovery efforts.
Page 16, line 36, must be clear that heritability doesn't actually indicate the "extent different traits are genetic", only the fraction of phenotypic variation explained by genetic variance.
For the last paragraph on page 17, are there any numbers that can further instill the point that it is "harder to obtain a sufficient number of samples required for accurate heritability estimates"? About how many samples/families would be needed to discern reliable heritability estimates from this approach, and how would this compare with numbers needed for methods based on GREML or LDSC?
Page 19, first paragraph, is it correct that SNP heritability is synonymous with narrow-sense heritability? Isn't narrow-sense heritability simply the heritability attribute to the additive effect of alleles (i.e. excluding dominance and interaction effects), whereas SNP heritability is the proportion of narrow-sense heritability accounted for by SNPs present on a genotyping microarray (along with the variants they tag)? Presumably indels and other variants poorly tagged on microarrays (or poorly imputed) contribute to narrow-sense heritability?
Page 20, second paragraph, is it true that residual population structure would only inflate h2_SNP and thereby decrease the difference between h2_SNP and h2_twin? In other words, this would certainly be a source of bias but not missing heritability per se.
Last sentence of page 22, not clear what is meant by "non-linear" phenotypes in this context.

**Authors’ response**

Brandes et al. have written an interesting and timely perspective on open problems in human genetics. Based on discussions with some colleagues, they have compiled a list of important problems that remain unsolved, either due to lack of data, appropriate methods, or lack of attention. While they make few points that are entirely novel and not observed in any previous literature, compiling the points in this format is valuable and insightful, and is a solid contribution to the literature. I can see this being particularly valuable for trainees that are trying to map out the frontiers of the field. The manuscript is well written and fun to read.

Reply: We thank the reviewer for the kind words and useful suggestions.

I have some comments - in the order of appearance - that are mostly suggestions of clarifications, some additional points, or references:

I find Table 1 to be too condensed to be helpful or more than a table of contents. I'd suggest making it more like Supplementary Table 1 where the essential points are summarized.

Reply: We thank the reviewer for this valuable feedback. We have merged most of the content of Supplementary Table S1 into Table 1 (while making it slightly more compact). Accordingly, Supplementary Table S1 is now removed.

"The required sample size is determined by the allele frequency and effect size of each association." - also by distribution/prevalence of the trait, especially for case-control traits where rare phenotypes require larger sample sizes.

Reply: Reading this sentence again, it seems not that important and out of context in the paragraph, so we have decided to just remove it (to avoid a premature, complicated discussion on all the factors affecting statistical power).

"Focusing on homogenous ethnic groups also underplays the potential genetic basis of phenotypic differences between groups." ..And excludes a large proportion of humans with more complex ancestries

Reply: Good point. We’ve added that to the sentence.

In the Population structure section, the authors use the word "ethnic" (identity/diversity/group) quite a lot. This may be appropriate depending on the exact meaning, but in the American context this has a rather specific use (Hispanic / non- Hispanic) that may confuse things. Also, it should not be used as ~synonymous to ancestry, and sentences such as "Individuals of European ancestry are overwhelmingly overrepresented in contemporary cohorts and, as a result, most published results do not directly transfer to other ethnic groups" sound somewhat problematic to me, contrasting European ancestry and other ethnic groups. The terminology around this issue is terribly confusing and somewhat controversial (see e.g. https://arxiv.org/abs/2106.10041 and https://www.nature.com/articles/s41588-021- 00952-6 ) but (thus?) I'd encourage the authors to take another look at this and make sure their terminology is at least consistent and as appropriate as possible. In many places, replacing "ethnic" with "ancestry/ancestries" might be better?

Reply: We thank the reviewer for bringing this terminology issue to our attention. We have removed the word “ethnicity” from the manuscript altogether, using instead “ancestry” or “population”, depending on the context. Open problem #7 has been renamed “ancestry diversity”, and we now use the term “cross-ancestry instead of “trans-ethnic”. We have left the word “ethnicity” only in one place in the manuscript where it still seems appropriate: “It is also a common practice to split cohorts by self- reported ethnic identities”, since questionnaires (e.g. in the UK Biobank) will still often ask study participants for their “ethnicity”.

"Like most genetic resources, haplotype reference panels are highly biased towards individuals of European ancestry, and so are the popular DNA microarrays (which usually genotype variants present in these populations). As a result, the genetic coverage obtained for non-Europeans is typically much lower" This is not untrue, but isn't this mostly a legacy issue? TOPMed imputation panels and e.g. the MEGA array are not that bad in terms of diversity. Is the coverage in current studies really MUCH lower for non-Europeans?

Reply: We have toned down this paragraph to indicate that this is still somewhat of an issue, but things are gradually improving.

"However, gene-based approaches are still susceptible to LD; it is possible that signal from nearby variants would leak into variants affecting the gene." Furthermore, it's not uncommon for expression of nearby genes to be coregulated - this is true biology of joint regulatory elements and would persist even if there was no LD leakage - and when that happens, multiple genes will be implicated even though only one might be causally related to disease. There's ~no way for genetics to disentangle this.

Reply: We have added the following sentence: “Moreover, even in the absence of LD, the expression of genes is often correlated, meaning that the association between the phenotype and the expression of a causal gene could leak into other genes”.

Regarding competitions and challenges for causal variant inference, this might be a good reference: https://onlinelibrary.wiley.com/toc/10981004/2019/40/9 - this has been done to some extent.

Reply: We have mentioned this and added the reference.

"could in fact be considered omnigenic, namely that they are affected by most of the genes in the genome [43]"...that are expressed in the trait-relevant tissue(s)

Reply: See our next response.

"In such cases, additional mapping of genomic loci associated with omnigenic traits, without considering other factors (such as effect sizes), is not expected to substantially contribute to our understanding of mechanism." - this, in the context of the preceeding section makes too strong claims, in my opinion. The omnigenic model is essentially a hypothesis that has received a fair amount of criticism and lacks empirical data that could provide the fundamental support/falsification for its assumptions. Even if true, they way to assign traits as omnigenic or not is not defined. I think some of these uncertainties should be reflected on here.

Reply: We have added the following clarification sentence: “While the term has also been used with reference to a specific mechanistic model suggested for such extreme polygenicity, here we use “omnigenic” simply to refer to extremely polygenic traits, irrespective of the mechanism”.

In the section "From causality of mechanism" the authors basically skip over the substantial amount of mechanisms-focused work that does not rely on perturbation experiments. I acknowledge that this is not the focus of the manuscript, but not mentioning efforts to annotate regulatory elements, causal genes, affected networks and relevant cell types of GWAS traits using data sets like ENCODE, GTEx and HCA is an oversight. This is essentially skipping over 15 years of functional genomics work to describe the next hot thing in town: perturb-seq (which is still largely untested as to its uses in GWAS interpretation).

Reply: We have added the following sentence: “Functional genomic annotations derived from experiments (and curated in resources such as ENCODE [55], GTEx [56] or HCA [57]) are also useful for suggesting mechanistic interpretations”.

Also, perturb-seq is a method to link gene knock-outs - not genetic variants - to gene expression. To my knowledge, there is no high-throughput perturbation approach (yet) "to detect variants that causally affect gene expression".

Reply: A work doing that was published quite recently (Ursu et al., 2020). We have added that reference.

"A useful practical approach to improving PRS performance is to incorporate clinical factors into the predictive models on top of genetic markers (e.g., using body mass index and birth weight to improve type-2-diabetes PRS [48])." My understanding is that while PRS alone can often work pretty well, one big practical challenge is that the marginal benefit over already used clinical predictors is modest. This may be worth mentioning in a clearer way.

Reply: We have added the following sentence: “However, as of today, PRS-based risk assessment generally provides only marginal benefit on top of clinical predictors already used”.

Transferability of PRS: I strongly recommend adding this reference here and in other applicable places: https://pubmed.ncbi.nlm.nih.gov/31999256/

Reply: We have added the reference and the following sentence to the paragraph discussing this open problem: “Even within the same dataset and ancestry group, prediction accuracy can vary based on characteristics such as sex, age or socioeconomic status”.

"but health providers would still want to see good evidence" -> health care providers

Reply: Fixed.

I find the heritability section to be interesting and well-written, but the first few pages of, discussing methods to estimate heritability, it reads largely like a review, rather than the topic of this manuscript: defining open problems in genetics. This content could be condensed (or re-used in another context) to highlight the open questions in the area rather than existing methods.

Reply: We are aware that this is not strictly the topic of the review and may seem like a digression (and that the review is quite long as it is), but we think that this quick introduction to heritability estimation (with a bit more formal terminology) is important to make the review accessible to a broad readership. Moreover, many of the pros and cons of the different methods come up in later parts of the review, so we think that this short introduction is helpful. Overall, the subchapter titled “Methods for estimating heritability” is one page long. Given these considerations, we are in favor of keeping it.

"In inbred mice, where the allele frequency of all variants becomes either 0% or close to 50% regardless of how rare they were in the original population..." I don't get why it would be 50%. Shouldn't it be 0% or 100%, i.e. one or the other allele is fixed in the inbred strains? Am I missing something? F1 crosses would have a lot of 50% variants but not inbred parents.

Reply: That was indeed a mistake in phrasing. We have fixed it and clarified that we are talking about “mice crossed from two inbred strains”.

"The recent release of 200K whole-exome sequences by the UK Biobank..." Now we already have genomes! :-) Please update.

Reply: Updated.

"Given what we know about complex biological systems, the burden of proof should be on those arguing for additive genetic effects being the primary source of heritability."

Do you indeed mean heritability, or missing heritability?

Reply: We mean heritability, not missing heritability. The additive genetic model assumes that most of the phenotypic variance attributed to genetics (i.e. the broad- sense heritability) could be explained by additive genetic effects. We see this as an independent problem to the missing heritability problem, at least in principle (of course there are many connections between them, as we point out throughout the review).

In the section about additive versus non-additive genetic effects, it might be worthwhile to mention the distinction between and discussion around statistical versus biological epistasis.

Reply: This is a very helpful distinction. We have mentioned it in the first paragraph of this chapter, and also in the chapter on epistasis, dominant and recessive effects.

Just a suggestion for the GxE part: autoimmune disease risk is a particularly compelling example of GxE being relevant for fundamental understanding of overall disease mechanisms, with rather compelling hypotheses and data pointing to a disease architecture where genetic variants predispose to inappropriate response to external microbial stimuli, and these jointly cause disease.

Reply: That’s a great idea. We have mentioned autoimmune disease as a general compelling example and added specific concrete examples (RA and oral bacteria, and MS and Epstein-Barr virus which was just published in Science).

Regarding the difficulty of finding GxE, it would be worth mentioning that functional analyses of cell state specific molecular effects of GWAS loci has been quite a powerful way to characterize GxE, even when the GxE effect may be difficult to detect at the GWAS level. In particular, response eQTL studies in immune cells have been important here.

Reply: We have added the following sentence: “In some cases, the study of GxE interactions can be informed by cellular experiments, if the organism-level exposures can be translated into cellular exposures that can be tested in the lab (e.g., cytokines as a proxy for inflammation)”.

"Just as the quality and representation of genotypes is expected to have a major influence on the results of genetic studies, so do the technicalities of how human phenotypes are defined and measured." - I think this is an issue of more than just technicalities; some of the problems of phenotyping (as discussed in the text) are actually very profound. I'd suggest dropping that word. Reply: We have rephrased the sentence and dropped this word, to make it clear that these are substantial and not just technical questions.

Reviewer #2

Summary/General comments:

In this manuscript by Brandes, Weissbrod, and Linial, the authors put forward a set of open questions in the field of human trait genetics. These questions span an expansive range of issues that include general issues (e.g. population structure, epistasis, GXE interactions), data-related issues, and specific challenges that persist in the field of human genetics. The authors address ongoing issues, biases, and limitations of genome-wide association studies (GWAS), heritability estimation, and polygenic risk scores with an aim of providing a helpful primer for geneticists, students, and researchers new to the field of human genetics. The authors propose a number of interesting questions and spotlight some areas that remain largely unaddressed - such as the underrepresentation of sex chromosomes in genetic discovery effots and the unexplored consequences of non-additivity in trait mapping and genetic prediction.

The scope of this manuscript is undoubtedly ambitious, and indeed is ripe with questions that are relevant to the field of human genetics and necessitate further exploration. However, there are areas where this manuscript can be substantially improved. The breadth of issues addressed is expansive, and at places seem disjointed which disrupts the narrative flow and readability. Moreover, the depth of content and discussion is lacking in certain subsections. I found some sections to be interesting and edifying (such as the sections on heritability and non-additivity), but others to be superfial ("direct vs indirect effects")ci. I think that all sections would benefit from additional vignettes that illustrate the problems and proposed solutions in greater detail. Too often, as I was reading through a section, I would be introduced to a problem, become interested in the problem, and then be introduced to a new problem in a different section without being sufficiently convinced or finding resolution in the previous section. The authors do provide citations at these points, but the manuscript would benefit from elaboration of specific examples or vignettes from these cited works, rather than passing references or one sentence descriptions. Moreover, there may be more coherent ways of organising the topics addressed in this manuscript that would elevate the readability and impact of this work. For example, by splitting it into two main sections that track along the two main applications introduced in the Scope section or, alternatively, by introducing broader themes and then having sub-sections corresponding to applications (for example, by introducing non-additivity at a high-level and then have subsections or vignettes that explore the consequences of non-additivity on heritability estimation, GWAS, and PRS). There are many interesting ideas presented in this manuscript and I believe that with further revision this could become a useful resource to the field.

Reply: We thank the reviewer for this feedback. We agree that some sections are more developed than others and that more examples would be helpful, but we feel that our review is already approaching that maximum length that would be appropriate, so there is a real tradeoff here. Given this tradeoff, we have made a conscious decision to gloss over some parts (and mostly relying on references) while delving into others topics in more detail. A key consideration in deciding how deeply to go into a topic was whether there already existed good references on the topic, as in the case of direct vs. indirect genetic effects (which we felt was quite adequately addressed in the review by Young, 2019). In other cases, such as our chapter on non-additivity, we felt there wasn’t adequate discussion of these topics in existing literature, so we decided to delve into it more deeply, hoping we could thereby provide additional value to the field. Throughout the review we attempted to provide simple and useful examples (when appropriate).

Major comments:

Writing seems needlessly verbose at various points. For example, in the sentence "The notion and tradition of publishing 'open problems' is borrowed from the mathematical disciplines, where the promotion and explicit discussion of major open challenges has a great role..". The words "notion", "promotion", and "great" seem superfluous as they don't substantively advance the argument. I found such long sentences to be a bit distracting and I think the readability of the manuscript would considerably improve with further revision.

Reply: We have tried to remove unnecessary adjectives and other needless words from the text throughout the review. In this specific example, we have simplified this sentence and removed the words “notion” and “promotion”.

On line 55 of Scope (page 5) you indicate that "functional elements in the human genome" is a topic "related to genetics but not to phenotypic variation in present-day humans". Yet, in the previous paragraph you indicate that a primary application is to "obtain insight into biological mechanism at the molecular and cellular level underlying the disease or trait under study". This seems to belie the extensive work based on integrating functional genomic information (e.g. enhancer elements, chromatin accessibility, hypo-methylated regions), which is often tissue- and cell-type specific, at non-coding loci associated with complex traits in order to resolve molecular mechanisms that influence phenotypic variation.

Reply: We have revised this paragraph to clarify that we do not ignore these topics, but rather that they are not our main focus (i.e. we don’t address open problems in these domains).

On line 35 of page 7 ("Genetic association studies and their limitations"), population structure and linkage disequilibrium are listed as the "two main issues limiting one from inferring a causal link".

These are certainly challenges that can confound association testing and complicate fine-mapping efforts, but I would think that the fact that most GWAS associations are non-coding would also be a main challenge in resolving a "causal link" between a variant and phenotype. More generally, inference of "causality" through association studies is not only complicated by sources of confounding (i.e. population structure) but also from a lack of biological plausibility (e.g. non-coding loci without any corroborating functional evidence).

Reply: That's a good point, but we chose to separate the discussion on statistical causality from that on biological explainability/interpretation (which is discussed in the chapter about mechanism).

Issues due to bias and chance (e.g. Winner's curse), reverse causality, and inconsistency of associations are also relevant.

Reply: We agree with the relevance of all of these problems, but we don’t think that they constitute major open problems in the field, unlike LD and population structure, as these issues are quite generic and don’t specifically apply to statistical genetics. We have revised the text to clarify that we see these two problems as the main open problems that are specific to genetic studies.

The problem of "residual population structure" is certainly interesting and provokes the question of how does one sufficiently control for population structure. How many PCs should one use in their GWAS? 5? 10? 20? It seems there is a lack of consistency in the field here that the authors could elaborate on further. A recent article by Florian Privé (Privé et al. 2020. Bioinformatics) proposes an interesting framework for generating and selecting PCs and applies it to UK Biobank data, indicating that an appropriate number of PCs is highly context specific.

Reply: This is indeed relevant to our very brief overview of PCA. We have added a sentence referencing this work.

The section on PRS could benefit from further elaboration on how PRS differs considerably from other standard risk factors (e.g. age, BMI, blood chemistry assays, etc.). For example, DNA, unlike other risk factors, does not change throughout one's lifespan (though interaction effects between PRS and other risk factors may differ temporally). It therefore need only be measured once. Moreover, a patient's genotype can be integrated with genetic information from many traits to derive many different PRS scores simultaneously.

Reply: These are all good points. We have added them to the third paragraph in the chapter on PRS.

In the second paragraph on page 17, it would be interesting to provide a description of LDSC in addition to GREML.

Also, the previous paragraphs seem to address broad-sense heritability, whereas these approaches for genomic data pertain to narrow-sense heritability, no? Isn't it important to differentiate between these two forms of heritability and discuss how this affects interpretation of heritability estimates from these approaches?

Reply: This is immediately discussed in the following chapter, which was now revised and titled “Types of heritability”.

Minor comments:

Not sure if "robust causal links" can be immediately gleaned from GWAS association results (Scope; page 5; line 38)

Reply: That’s true, but we really try to keep the Scope chapter as short and concise as possible (so that readers won’t get tired and bored before they actually start reading the review). Therefore, we think it’s best to leave the discussion on the limitations of GWAS in inferring robust causal links to the first chapter discussing association studies.

On lines 56-58 (page 5, Scope), in what sense would Mendelian Randomization be considered a method that "uses genetics only" to study relationships between traits, but not GWAS?

Reply: We’ve modified the text to properly explain it: “We also do not discuss methods that use genetics only as an instrument to study relationships between traits, such as Mendelian Randomization (which attempts to find out through genetic evidence whether one trait affects another, for example whether high cholesterol levels increase the risk for heart disease)“.

Page 6, line 8, Scope, it doesn't seem accurate to say that Mendel's first law dictates "pure dominant or recessive inheritance with high penetrance", as the first law (i.e. law of segregation) merely states that two copies of each hereditary factor (i.e. allele) are separated during the production of gametes. The first law does not explicitly address modes of inheritance or penetrance.

Reply: We have rephrased the sentence and no longer refer to it as the first law.

Given that two of the three target audiences for this manuscript, as described in the letter to the editor, are early stage students and researchers new to the field of statistical genetics, it may be useful to provide high-level descriptions of PCA (and perhaps LMMs) as they are important for contemporary GWAS but may not be familiar to the uninitiated.

Reply: That could indeed be useful for trainees, but we don’t think that this review would have an added value in describing such general-purpose algorithms (and the manuscript is already very long). Also, it is our impression that these topics are already well covered by existing scientific literature and informal training materials.

Page 8, fourth paragraph, it seems that family-based trio studies do not directly "account for population structure" but rather obviate the problem altogether. I do find the proposal that incorporating both related and unrelated together in the same study interesting as described in the beginning of page 9. Can you provide an in-text example of how this strategy has been used to improve trait mapping/ GWAS power?

Reply: We have slightly revised the text and added a specific example: “an analysis of individuals from the UK Biobank, some of which are family relatives, showed an increase in statistical power compared to a filtered dataset of unrelated individuals (beyond the increase attributed to the larger sample size)”.

I'd also cite the TOPMED study in addition to the HRC in the section on imputation.

Reply: We have mentioned TOPMed alongside HRC.

Probably worth mentioning that the lack of non-European representation in genetic studies immediately undermines imputation quality of non-European genotyping data.

Reply: We have revised the text, which now reads: “Like most genetic resources, haplotype reference panels and DNA microarrays are still somewhat biased towards individuals of European ancestry (although improvements have been made [33]). As a result, the genetic coverage and quality of genotype imputation are typically lower for non-Europeans (open problem #7: ancestry diversity) [14]”.

Page 11, third paragraph, overall this paragraph is vague. Would be better to either elaborate on what the "powerful tools" and questions of interest are, and the specific biases, or simply remove this paragraph.

Reply: For the sake of clarity and shortening the manuscript, we have decided to remove this paragraph.

Seems that the section of direct vs indirect effects may also benefit from a discussion of pleiotropy which can certainly complicate the aim of resolving causal mechanisms between GWAS variants and phenotypes.

Reply: We have added a short paragraph on pleiotropy at the end of the chapter on mechanism.

According to Boyle et al 2017, the key insight from the "omni-genic" model is not that complex traits are "affected by most of the genes in the genome", but rather that polygenicity can be understood as an interplay between a subset of core genes influencing expansive gene regulatory networks, and many peripheral variant effects on genes outside the set of core genes. Hence, the heritability may involve the effects of many variants (akin to polygenicity), but only a subset of genes will be relevant to causal mechanisms and drug target discovery efforts.

Reply: We have added the following clarification sentence: “While the term has also been used with reference to a specific mechanistic model suggested for such extreme polygenicity, here we use “omnigenic” simply to refer to extremely polygenic traits, irrespective of the mechanism”.

Page 16, line 36, must be clear that heritability doesn't actually indicate the "extent different traits are genetic", only the fraction of phenotypic variation explained by genetic variance.

Reply: We have fixed this mistake and rephrased the sentence.

For the last paragraph on page 17, are there any numbers that can further instill the point that it is "harder to obtain a sufficient number of samples required for accurate heritability estimates"?

About how many samples/families would be needed to discern reliable heritability estimates from this approach, and how would this compare with numbers needed for methods based on GREML or LDSC?

Reply: We apologize for the lack of clarity. We were simply referring to the difficulty of recruiting families, not to lower accuracy given a certain sample size. We have rephrased the text to clarify that.

Page 19, first paragraph, is it correct that SNP heritability is synonymous with narrow- sense heritability? Isn't narrow-sense heritability simply the heritability attribute to the additive effect of alleles (i.e. excluding dominance and interaction effects), whereas SNP heritability is the proportion of narrow-sense heritability accounted for by SNPs present on a genotyping microarray (along with the variants they tag)? Presumably indels and other variants poorly tagged on microarrays (or poorly imputed) contribute to narrow-sense heritability?

Reply: Our definitions were indeed somewhat inaccurate. We have rewritten this entire small chapter (now titled “Types of heritability”) to better reflect the nuances of broad- sense vs. narrow sense vs. SNP heritability.

Page 20, second paragraph, is it true that residual population structure would only inflate h2_SNP and thereby decrease the difference between h2_SNP and h2_twin? In other words, this would certainly be a source of bias but not missing heritability per se.

Reply: It is correct that a bias could generally go both ways, but in the case of unaccounted population structure, shared environmental factors (within fine-grained ancestry groups) are confounded with genetic factors, meaning that the overall variance estimated to be attributed to genetics could be larger than its true value, i.e. an inflation of h2_SNP. Even if there are doubts whether or not this is substantial, it’s a plausible enough hypothesis of a component of the missing heritability problem, so it should be taken seriously until proven otherwise.

Last sentence of page 22, not clear what is meant by "non-linear" phenotypes in this context.

Reply: We have revised it into “phenotypes that cannot be approximated well by a linear model”.
